# The Association Between Neurocognitive Disorders and Gustatory Dysfunction: A Systematic Review and Meta-Analysis

**DOI:** 10.1007/s11065-023-09578-3

**Published:** 2023-02-20

**Authors:** Elisa Mantovani, Alice Zanini, Maria Paola Cecchini, Stefano Tamburin

**Affiliations:** 1 Department of Neurosciences, Biomedicine and Movement Sciences, Neurology Section, University of Verona, Piazzale Scuro 10, I-37134 Verona, Italy; 2 Department of Neurosciences, Biomedicine and Movement Sciences, Anatomy and Histology Section, University of Verona, Strada Le Grazie, 8, I-37134 Verona, Italy

**Keywords:** Neurocognitive disorders, Taste, Biomarkers, Systematic review, Meta-analysis

## Abstract

Olfactory and gustatory dysfunction have been reported in mild and major neurocognitive disorders (NCDs), with variable results. While olfactory dysfunction has been consistently explored, reports on gustatory alterations are limited. We systematically reviewed case-control studies evaluating gustatory function in NCDs with various etiologies and different neuropathology. Eighteen studies were included in the systematic review, and eight were included in the meta-analysis. Most studies were on Alzheimer’s disease (AD) and Parkinson’s disease (PD). Pooled analyses showed worse global taste threshold and identification (sour in particular) scores in AD than controls and worse global, sweet, and sour scores in AD compared to mild cognitive impairment (MCI). PD with MCI showed worse global, sweet, salty, and sour scores than controls and cognitively unimpaired PD. Taste dysfunction was differentially associated with the severity of cognitive deficits. Gustatory dysfunction may represent a potential cross-disease chemosensory biomarker of NCD. Whether gustatory alterations may be a pre-clinical biomarker of NCD requires further studies.

**Supplementary Information:**

The online version contains supplementary material available at 10.1007/s11065-023-09578-3.

## Introduction

### Neurocognitive Disorders: Classification, Neuropathology, and Biomarkers

Neurocognitive disorders (NCDs) encompass a series of acquired manifestations including delirium, minor NCD, which corresponds to mild cognitive impairment (MCI; Petersen et al., 2011), and major NCD (i.e., dementia), which results in a significant decline in cognitive functioning from a previously attained level of performance (American Psychiatric Association, 2013). The framework proposed in the Diagnostic and Statistical Manual of Mental Disorders – 5th edition is based on clinical presentation, but mild and major NCDs can be classified according to different etiologies, with Alzheimer’s disease (AD), vascular dementia (VaD), Parkinson’s disease (PD), frontotemporal degeneration (FTD), traumatic brain injury, infections, and alcohol abuse representing common causes (Table S1; see also Sachdev et al., 2014).

Neurocognitive disorders, especially those due to neurodegenerative processes, are associated with many brain alterations, some of which overlap between different etiologies. Early anatomical changes involving medial temporal lobe structures (i.e., entorhinal cortex and hippocampus), have been reported to differentiate MCI with AD biomarkers and early AD from healthy subjects (Talwar et al., 2021). With disease progression, additional structures are affected in AD, including the amygdala, olfactory tract, cingulate gyrus, and thalamus, and atrophy spreads to cortical regions, with frontal, parietal, and temporal cortices being more involved (Chandra et al., 2019). The anterior insula and cingulate cortex are the first brain regions that show structural and metabolic neuroimaging abnormalities in FTD, with the additional involvement of the frontal poles, dorsolateral and medial prefrontal cortices, orbitofrontal, and premotor cortex as the disease progresses (Peet et al., 2021). Transentorhinal and forebrain (e.g., hypothalamus, thalamus, limbic system) regions, anterior dorsal insular cortex, orbitofrontal, prefrontal, and posterior cingulate cortices are also affected in PD and other parkinsonisms related to α-synucleinopathy (Saeed et al., 2020; Bidesi et al., 2021).

The most definitive classification system for NCDs is based on the underlying neuropathology, which, in turn, is categorized largely according to the observed accumulation of abnormal protein aggregates in neurons and glia, with the vast majority of non-VaD cases falling into six main categories of neurodegenerative proteinopathy, including amyloid-beta (Aβ), microtubule-associated protein tau, TAR DNA-binding protein 43 (TDP-43), fused in sarcoma, α-synuclein, and prion protein (Elahi & Miller, 2017; Kovacs, 2019).

The diagnosis of NCDs is usually *post-mortem*, but biomarkers may offer important *in-vivo* information since the early NCD stages, such as neuropathological changes, may begin decades before the onset of clinical features. NCD biomarkers provide diagnostic and prognostic information and might be helpful for future disease-modifying treatment strategies (Aarsland et al., 2021; Hansson, 2021; Stefani et al., 2021). Similar clinical pictures may be related to different neuropathological processes, and clinical diagnosis may not offer information on the underlying proteinopathy. In addition, multiple brain pathologies (i.e., Aβ, tau, α-synuclein, TDP-43) may overlap across several neurodegenerative disorders in the elderly, thus further complicating neuropathological-based diagnosis (Karanth et al., 2020). Following these lines of reasoning, the traditional diagnosis of probable AD based on clinical and instrumental criteria (McKhann et al., 1984), has been reiterated in the more recent description of probable AD with proven pathophysiological processes based on biomarkers (Albert et al., 2011; McKhann et al., 2011). A further step on the use of biomarkers in AD is the very recent AT(N) classification system developed by the National Institute on Aging and Alzheimer’s Association Research Framework (Jack et al., 2018). The AT(N) classification system allows a more nuanced staging of patients into syndromes (i.e., cognitively unimpaired; MCI; dementia) and a numerical clinical staging of AD (i.e., stages 1–6) according to clinical evaluation and Aβ deposition (A), pathologic tau accumulation (T) and neurodegeneration (N) (Jack et al., 2018). Fluorine-18-fluorodeoxyglucose, Aβ and tau positron emission tomography imaging and cerebrospinal fluid measures of Aβ fractions, tau and phospho-tau are widely diffused diagnostic biomarkers for AD. Nevertheless, testing for other proteinopathies is available in a few centers (e.g., α-synuclein) or not applicable in the clinical setting (e.g., TDP-43) (Sheikh-Bahaei et al., 2017; Hansson, 2021).

Despite recent advances in this field, most imaging and bio-fluid biomarkers are either expensive or invasive, thus restricting their potential applicability in clinical and research practice (Hansson, 2021). Blood-based biomarkers, although providing straightforward and non-invasive collection methods, require complex processing techniques that are not widely available (Ashton et al., 2020). Additional valid, minimally invasive, and cost-effective biomarkers for NCDs are therefore required.

### Gustatory Function: Anatomy and Assessment

The gustatory system detects, identifies, and establishes the palatability of food and beverage through taste receptor cell activation. Gustatory information is then conveyed by the taste nerves (i.e., facial, glossopharyngeal, and vagus) to the central nervous system. Somatosensory information (i.e., touch, temperature) via trigeminal and glossopharyngeal nerves, together with smell and visual stimuli, contribute to the full flavor experience (Shepherd, 2006; Cecchini et al., 2015).

**Fig. 1:**
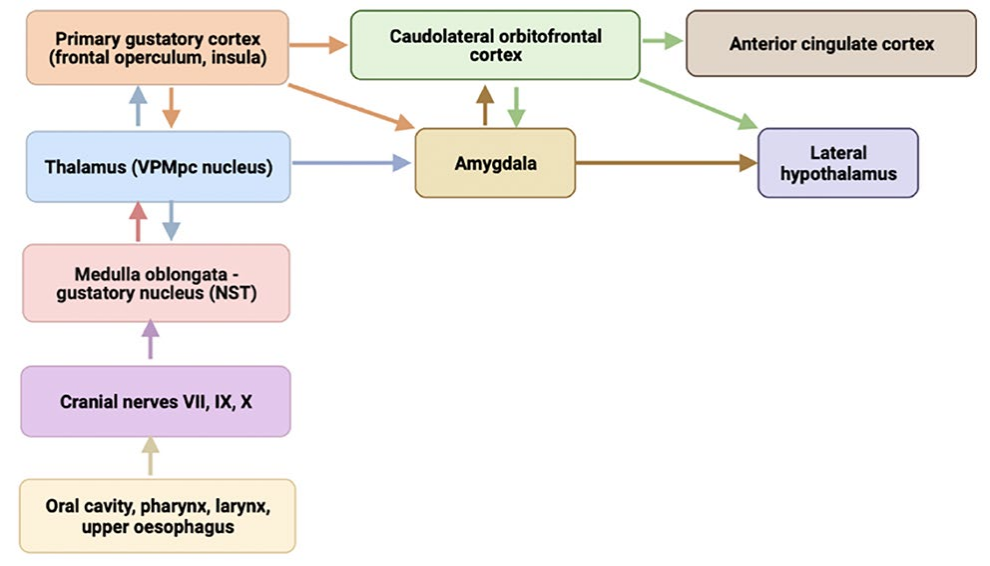
Schematic diagram illustrating the anatomy of the main gustatory processing. List of abbreviations: NST = nucleus of the solitary tract; VPMpc = ventral posteromedial nucleus of the thalamus, parvocellular part

The anatomy of the gustatory system is complex, and a wide mucosal surface encompassing the oral cavity, pharynx, larynx, and upper esophagus is involved in chemosensory perception so that taste can be considered a robust sense (Bartoshuk, 1989; Cecchini et al., 2018). Information from the taste receptor cells is first transmitted via cranial nerves to the gustatory nucleus, that is, the rostral division of the nucleus of the solitary tract (NST) in the medulla oblongata. Before reaching brain cortical areas, NST fibers project to the ventral posteromedial nucleus of the thalamus, parvocellular part. Thalamic neurons project to the primary gustatory cortex (i.e., frontal operculum, insula) involved in taste identification and memory, which in turn sends afferent information to other areas, for example, the multimodal orbitofrontal region and the anterior cingulate cortex. Additional subcortical areas are involved in gustatory processing (e.g., the lateral hypothalamus, mainly involved in the modulation of satiety). On this matter, it is important to mention that taste and smell express a remarkable hedonic quality, which has a significant psychological impact (Bochicchio & Winsler, 2020). The hedonic value of taste is represented in different areas of the brain and probably also in the amygdala, which is thought to be involved in the representation of emotional states. This notwithstanding, the role of the amygdala in the representation of the hedonic or emotional value of smell and taste is still debated (Anderson & Phelps, 2002; Soudry et al., 2011). Furthermore, cortical gustatory areas send efferent projections to the NST and other subcortical areas for top-down modulation of gustatory afferents (Fig. 1) (Simon et al., 2006; Iannilli & Gutziol, 2019; Vincis & Fontanini, 2019).

In humans, five basic taste qualities can be described, including sweet, salty, sour, bitter, and umami taste. Besides, qualities such as fat, metallic, and carbonation were reported as putative additional basic tastes (Chandrashekar et al., 2009; Chaudhari & Roper, 2010). Umami, known as the fifth taste, is typically elicited by monosodium glutamate, some aminoacids, or by purine nucleotides, and is generally not included in routine gustatory tests (Kurihara, 2015). Umami taste was found to be hard to conceptualize by the European population, even if monosodium glutamate is found in a wide range of foods and flavor enhancers (Landis et al., 2009; Cecchini et al., 2019a).

Gustatory testing is an important step in the assessment of gustatory disorders, since the accuracy of self-reported taste impairment is poor (Soter et al., 2008; Oleszkiewicz & Hummel., 2019). Gustatory disorders are defined as either qualitative, including dysgeusia (i.e., taste distortion or taste perception in the absence of a gustatory stimulation), which is reported by patients but not quantitatively assessed, or quantitative, including ageusia (i.e., complete loss of taste), hypogeusia (i.e., diminished taste), and hypergeusia (i.e., increased taste sensitivity), which can be measured but often not reported by patients. In clinical practice, isolated gustatory deficit is uncommon, and complete ageusia is very rare, with most patients generally presenting combined olfactory and gustatory impairment (Welge-Lüssen et al., 2011). In this regard, it is important to distinguish between taste and flavor, the latter being among the most complex and powerful human sensations. Flavor perception is due to retronasal stimulation during food ingestion, when volatile molecules released from the food in the mouth are conveyed, through the movements of the mouth, from the back of the oral cavity up through the nasopharynx toward the olfactory epithelium. Because the volatile molecules arise in the mouth during eating, the sensation is perceived as originating within the mouth. Thus, this retronasal food-derived sensation is normally judged to be part of the “taste” of the food. Hence, although a great part of flavor is due to smell, flavor is often attributed to “taste” (Shepherd, 2006; De Rosa et al., 2019). Indeed, a referred gustatory deficit usually reflects a loss of flavor perception as a function of smell loss, rather than a true taste impairment, because flavor and taste follow different neuronal pathways. For these reasons, olfaction and taste must be assessed separately and by validated methods (Fark et al., 2013). Gustatory assessment may include different psychophysical tests using chemical stimuli to measure the ability to perceive the basic taste qualities or using electrical stimuli (i.e., weak anodal electric current). The latter method is generally considered a sensitive and rapid measure of gustatory threshold and is very popular in Japan (Ikeda et al., 2005). Chemical and electrical taste tests can be further divided into whole-mouth and regional tests (see Table S2 for details). Various factors including geographical and methodological differences could influence the test results, thus findings reported in the literature should generally be interpreted with caution (Welge-Lüssen et al., 2011).

### Gustatory Dysfunction: A Potential Biomarker of Neurocognitive Disorders?

Olfactory abnormalities have been associated with several NCDs of different severity and etiology (Thompson et al., 1998; Haehner et al., 2009; Bathini et al., 2019; Doty & Hawkes, 2019). Various studies have indeed reported olfactory impairment in patients with MCI and dementia (Roalf et al., 2017; Bathini et al., 2019). Olfactory dysfunction has been associated with cognition in AD and PD from preclinical stages (Roberts et al., 2016; Doty & Hawkes, 2019). Several lines of evidence suggest a close relationship between neurodegenerative disorders and olfactory dysfunction. The olfactory system, particularly the anterior olfactory nucleus, may be involved early in AD and PD neuropathology (Ubeda-Banon et al., 2020). Poorer olfaction has been associated with structural abnormalities of the peripheral olfactory system and the primary olfactory cortex, which are affected early by α-synucleinopathy (Brozzetti et al., 2020), and the hippocampus and the entorhinal cortex (Growdon et al., 2015), which are the first brain areas to undergo neurodegeneration in AD.

Brain regions involved in gustatory processing (i.e., orbitofrontal cortex, cingulate gyrus, multimodal integrative areas, amygdala, hippocampus, and other areas within the limbic system) are affected in MCI, AD, and PD-dementia (Sewards, 2004; Lang et al., 2006; Doty & Hawkes, 2019). In line with these anatomical data, slight gustatory impairment has been documented in some NCDs, but results are sparse and often diverging, with gustatory function being reported either as deteriorated or spared according to disease severity (Cecchini et al., 2015; Doty & Hawkes, 2019). Central mechanisms (i.e., decrease in the taste information processing) rather than peripheral mechanisms (i.e., impaired transmission from sensory receptors) have been proposed to explain these discrepancies (Kouzuki et al., 2020). Despite the overlap between brain areas involved in taste and cognition, the reports on gustatory function in NCDs are far more limited than those on smell, probably because taste assessment is rarely performed in the clinical setting, and because taste might be more resistant to injury being conveyed by three cranial nerves per side (Doty et al., 2018).

The association between NCDs and altered gustatory function is still unclear, so it might be clinically informative to better investigate this relationship. Unresolved questions include (a) whether patients with NCDs show poorer gustatory function than cognitively intact controls, (b) whether the severity of NCDs is associated with changes to taste (as psychophysical tests need patient cooperation), (c) whether different features of taste sensitivity (i.e., threshold, identification, intensity) could be differentially involved in NCDs, and (d) whether NCDs due to different etiologies are associated to specific patterns of gustatory dysfunction (e.g., overall impairment, dysfunction of specific taste qualities perception).

To answer these questions, this paper has two aims, namely, (a) to systematically review studies evaluating gustatory function with validated tests in NCDs due to various etiologies, and (b) to meta-analyze data on the association between cognitive impairment and gustatory dysfunction.

## Methods

The systematic review and meta-analyses were conducted according to the Preferred Reporting Items for Systematic Reviews and Meta-Analyses (PRISMA) recommendations (Moher et al., 2015; Page et al., 2021). The research protocol was registered in the International prospective register of systematic reviews PROSPERO (registration number: CRD42022314545; Mantovani et al., 2022).

### Eligibility Criteria

Case-control studies published in peer-reviewed journals (languages included English, Italian, French, and German) that assessed gustatory function with at least one validated chemosensory assessment method, using either chemical or electric stimuli, in patients with confirmed cognitive dysfunction of different severity (i.e., mild, major NCDs) and various etiologies (i.e., AD, VaD, Lewy body dementia, LBD, PD, FTD, multiple etiologies) were considered eligible and therefore included in the systematic review. No restrictions were placed in terms of study design or publication date. Case reports, reviews, letters, commentaries, abstracts, conference proceedings, and studies reporting only non-validated methods were excluded.

The SPIDER framework (Cooke et al., 2012) of the study is reported in Table 1.

**Table 1:** SPIDER criteria (Cooke et al., 2012)

**S**ample	Patients with neurocognitive disorders of different severity (i.e., minor, mild cognitive impairment; major, dementia) due to various etiologies (i.e., Alzheimer’s disease, vascular dementia, Parkinson’s disease, parkinsonism, frontotemporal dementia, multiple and/or mixed etiologies)
**P**henomenon of **I**nterest	The association between neurocognitive disorders and gustatory dysfunction
**D**esign	Case-control studies
**E**valuation	Taste function outcomes (i.e., threshold-detection, threshold-recognition, identification, intensity)
**R**esearch type	Qualitative and/or quantitative methods

### Search Strategy

The PubMed, Web of Science, and Science Direct search engines were consulted on October 10th, 2020 for peer-reviewed papers published from database inception until September 30th, 2020 with the following search string: (taste OR gustation OR gustatory) AND (neurocognitive disorders OR dementia OR mild cognitive impairment OR cognition OR cognitive function). The search was updated on June 20th, 2021 to ensure currency of results.

### Study Selection

Search results were uploaded to Rayyan software, a web-based app to facilitate collaborations among reviewers during the study selection phase, by supporting them throughout the entire systematic review process (i.e., merging of records from multiple search engines, duplicates identification, title and abstracts screening, full-texts screening, inclusion and exclusion decisions according to eligibility criteria, identification of conflicts between reviewers) (Ouzzani et al., 2016). Two authors (EM, AZ) independently screened titles and abstracts to identify relevant studies to undergo full-text inspection, which was performed in a double-blind fashion. Moreover, the reference lists of relevant papers (i.e., previously published narrative, systematic reviews or meta-analysis found through the literature search but discarded in consideration of the study design, and studies fulfilling the eligibility criteria and therefore included) were manually inspected for any additional study potentially missed in the databases search. Any disagreement was planned to be solved by consensus between the two independent reviewers or, in case of persistent disagreement, by consulting two other authors (MPC, ST).

### Data Collection

Two authors (EM, AZ) independently extracted the following data from the included papers, using a shared, previously pilot-tested collection form to ensure consistency during the process, including population (i.e., age, gender, sample size), underlying neuropathological condition (i.e., AD, VaD, LBD, PD, FTD, multiple etiologies), neuropsychiatric comorbidity (i.e., anxiety, depression), cognitive dysfunction severity, cognitive testing (i.e., screening test, single cognitive domains), gustatory testing, main results. For studies on PD, the following additional clinical features were extracted: disease duration, motor or non-motor symptoms presence and severity, levodopa equivalent daily dose (LEDD).

### Risk of Bias and Study Quality

Two authors (EM, ST) independently assessed risk of bias and study quality with the case-control quality assessment instrument provided by the Newcastle-Ottawa Scale (NOS) (Wells et al., 2013). Selection, comparability, and exposure domains were assessed, and each study could be awarded a maximum total score of 9. Scores between 0 and 3, 4–6, and 7–9 were considered as suggesting low, moderate, or high quality, respectively.

### Data Analysis

A systematic and descriptive analysis of the results was provided in the text and tables to summarize the characteristics and findings of the included studies.

Separate meta-analyses were performed for studies assessing gustatory function in patients with NCDs due to AD and PD. Data from patients with NCDs due to other etiologies (e.g., VaD, FTD) did not undergo meta-analysis because of the high heterogeneity of the samples and the absence of separate data for etiological subtypes. Original datasets were obtained from the corresponding authors of two studies (Masala et al., 2020; Nigam et al., 2021), and data for PD patients with MCI versus those without MCI were calculated as secondary analysis. Data were analyzed using Review Manager (RevMan version 5.4, The Cochrane Collaboration). Following the Cochrane Handbook recommendations, mean difference (MD) was chosen as effect size measure since the outcomes of interest were all reported using the same tools (i.e., Taste Strips Test, TST; Filter Paper Disc, FPD) for each meta-analysis (Borenstein, 2009a). MD was calculated from reported means and standard deviations of taste quantitative outcomes (overall test score; single taste qualities, i.e., sweet, salty, acid, bitter) that were extracted separately for cases and controls. The Cochran’s *Q* test, Higgins I^2^, along with the tau-squared (τ^2^) statistics were used to quantify heterogeneity between studies. The Cochran’s *Q* test assesses the total heterogeneity across all effect sizes. A significant *Q* value suggests that the true effects vary, thus indicating larger variations across studies. I^2^ measures the proportion of the variability in point estimates that can be attributed to heterogeneity (Higgins & Thompson, 2002; Higgins et al., 2003). Despite its common use and being more intuitive (i.e., there are widely used benchmarks for its interpretation), I^2^ is a relative measure that heavily depends on the precision of the included studies and is proportional to the study size. In contrast, τ^2^ statistics is a real point estimate of the magnitude of heterogeneity and insensitive to the precision of the included studies. τ^2^ may be a more informative measure of heterogeneity and can be used with I^2^ to provide more reliable information (Borenstein, 2009b; Borenstein et al., 2017). As the included studies were quite heterogeneous in terms of population and outcome measures, random-effect models were applied.

Publication bias was planned to be performed through the inspection of funnel plots. Sensitivity and moderator analyses were planned to be performed depending on the results on the heterogeneity and the number of the included studies per outcome, respectively (Borenstein, 2009b). The level of statistical significance was set at 5%, and 95% confidence intervals (CIs) were calculated. The results were displayed graphically using forest plots.

## Results

### Identification and Selection of the Studies

**Fig. 2:**
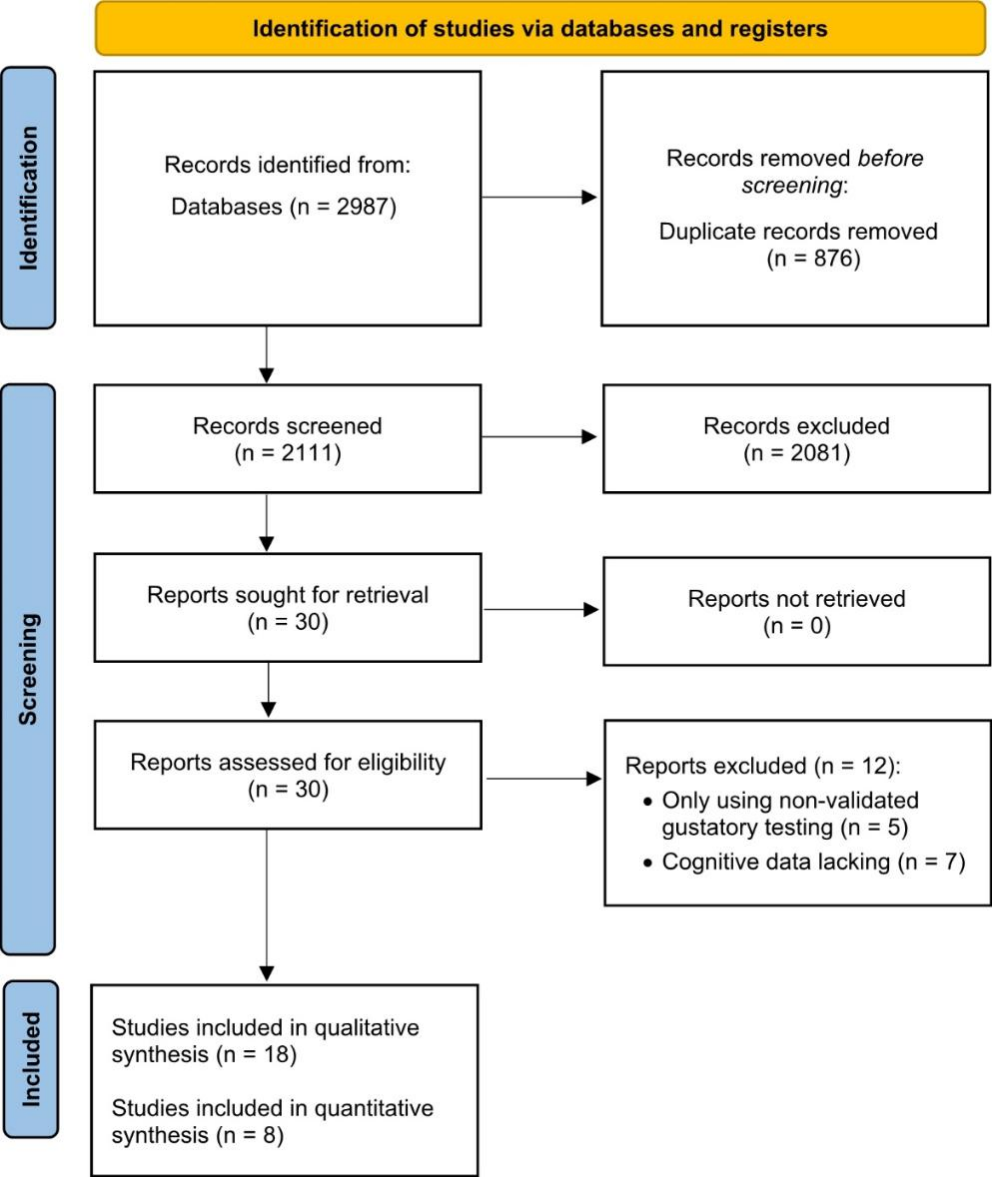
PRISMA diagram of the study (Page et al., 2021; http://www.prisma-statement.org). *From*: Page MJ, McKenzie JE, Bossuyt PM, Boutron I, Hoffmann TC, Mulrow CD, et al. The PRISMA 2020 statement: an updated guideline for reporting systematic reviews. BMJ 2021;372:n71. doi: 10.1136/bmj.n71.

A total of 2,987 records were identified through literature search. After duplicates removal, 2,111 records were screened through titles and abstracts and 30 papers were obtained for full-text screening. Two authors (EM, AZ) independently evaluated the 30 selected papers for in-depth examination. Disagreement concerned three papers (inter-rater agreement = 90%) and was solved by consulting two other authors (MPC, ST). Eighteen studies fulfilled the inclusion criteria and were therefore included in the systematic review, and eight out of eighteen studies were also included in the meta-analysis (Fig. 2).

Studies were grouped according to the etiology of cognitive dysfunction (i.e., AD, N = 10; PD, N = 3; other or mixed etiologies, N = 5). The studies on AD were further divided into two categories, depending on the type of MCI or dementia diagnosis (i.e., clinical, clinical and biomarkers).

### Studies on Patients with Neurocognitive Disorders due to Alzheimer’s Disease

Ten studies assessed gustatory dysfunction in patients with AD (Murphy et al., 1990, 1999; Steinback et al., 2010; Sakai et al., 2017; Ogawa et al., 2017; Kouzuki et al., 2018, 2020; Churnin et al., 2019; Contri-Degiovanni et al., 2020; Doorduijn et al., 2020) (Table 2).

**Table 2:** Studies on patients with neurocognitive disorders due to Alzheimer’s disease (AD)

Ref.	Population	Diagnostic criteria for AD	Neuropsychiatric comorbidity	Cognitive dysfunction severity	Cognitive testing		Gustatory testing (taste function assessed)	Results^§^
					Screening	Tested domains		
*Diagnosis based on clinical criteria only (i.e., no biomarkers confirmation); MCI + AD*								
Murphy et al. 1990	Patients: 20 (M: 10, W: 10; age: 72.7 ± 7.0) Controls: 20 (M: 10, W: 10; age: 72.3 ± 5.4)	AD (NINCDS-ADRDA criteria, 1984)	NA	MMSE: 20.6 ± 4.0 IMC: 13.6 ± 6.0 DRS: 105.7 ± 17.7	MMSE, IMC, DRS	Memory, language, attention, visuospatial, problem-solving abilities	Sweet threshold: two-alternative, forced-choice, staircase procedure (DT)	No difference between AD and controls
Murphy et al. 1999	Patients: 78 (age: 72.9 ± 7.8) Controls: 78 (age: 73.4 ± 7.6)	Probable AD (NINCDS-ADRDA 1984 and DSM-III-R criteria)	NA	DRS very mild AD: 137.4 ± 3.5 DRS mild AD: 121.1 ± 4.1 DRS moderate AD: 106.5 ± 5.3	MMSE, IMC, DRS	Memory, attention, abstraction/problem solving, motor, verbal/language, perceptual/constructional, orientation	Sweet threshold: two-alternative, forced-choice, staircase procedure (DT)	No difference between groups for taste thresholds
Steinbach et al. 2010	Patients: 59 (M: 30, W: 29; 72.5 ± 7.7) Controls: 29 (M:12, W:17; age: 68.2 ± 3.9)	MCI (Petersen criteria, 2001): 29 AD (NINCDS-ADRDA criteria, 1984): 30	Depression (use of antidepressant drugs)	MMSE MCI: 26.8 ± 2.2 MMSE AD: 21.7 ± 4.7	MMSE	NA	Taste Strips Test (sweet, sour, salty, bitter) (ID) Subjective questionnaire on taste function	MCI and AD patients scored worse than controls for the Taste Strips Test (i.e., overall and single taste quality scores) *Subjective perception of taste function was better for controls than for MCI and AD*
Sakai et al. 2016	Patients: 32 (M: 13, W: 19; age: 70.0 ± 8.3) Controls: 22 (M: 9, W: 13; age: 67.8 ± 7.0)	Probable AD (NINCDS–ADRDA criteria, 1984 and MRI findings)	Behavioral and psychiatric symptoms (NPI score)	CDR 0.5: 11 CDR 1: 15 CDR 2: 6	MMSE	Memory, premorbid intellective ability, executive function	Filter Paper Discs Test (sweet, salty, sour, bitter) (DT + RT) Taste discrimination task (sweet, salty, sour, bitter)	Total gustatory threshold values for DT and RT were higher in AD than controls Higher DT for sweet, salty, bitter and RT for sweet and sour in AD than controls *No difference between AD and controls for taste discrimination task*
Ogawa et al. 2017	Patients: 22 (M: 9, W: 19, age: 84.0 ± 6.0) Controls: 49 (M: 13, W: 36; age: 71.0 ± 8.3)	AD (DSM-IV TR criteria)	NA	MMSE: 17.5 ± 3.8	MMSE	NA	Filter Paper Discs Test (sweet, salty, sour, bitter) (DT + RT) Electrogustometer (DT)	AD showed reduced DT and RT for all the four basic taste qualities in the anterior and posterior tongue using the Filter Paper Disk Test than controls
Churnin et al. 2019	1367 older adults, age ≥ 60 years	MCI (based on CERAD total score) AD (based on CERAD total score)	NA	CERAD total score MCI: < 6.5 CERAD total score dementia: < 4.5	NA	Learning and memory, attention, executive function	NIH Toolbox (salty, bitter) (ID)	Inability to recognize salty taste quality was associated with dementia
Contri-Degiovanni et al. 2020	Patients: 60 (age: 79.6 ± 6.8) Controls: 30 (age: 77.0 ± 5.8)	AD (DSM-IV criteria)	NA	CDR 1: 37 CDR 2: 23	MMSE	NA	Taste Strips Test (sweet, salty, sour, bitter, umami) (ID)	Moderate AD patients showed worse overall, bitter and salty taste identification than age-matched controls and worse sweet taste identification than mild AD patients
Kouzuki et al. 2020	Patients: 72 (M: 24, W: 48; age: 81.3 ± 2.1) Controls: 14 (M: 3, W: 11; age: 74.5 ± 1.7)	MCI (Petersen criteria, 2011) AD (DSM 5 criteria)	NA	TDAS MCI: 7.0^†^ (5–12)* TDAS AD: 18.0^†^ (11–23)*	TDAS	NA	Japanese whole mouth procedure (sweet, salty, umami, sour, bitter) (DT + RT)	Taste RTs were higher for AD compared to controls
*AD diagnosis based on clinical and biomarkers criteria; MCI + AD*								
Kouzuki et al. 2018	Patients: 74 (M: 22, W: 52; age: 79.4 ± 1.2) Controls: 40 (M: 10, W: 30; age: 76.0 ± 1.1)	aMCI (Petersen criteria, 2011) AD (DSM-IV criteria)	NA	ADAS-j cog aMCI: 8.9 ± 0.4 ADAS-j cog AD: 16.3 ± 1.0 TDAS aMCI: 7.3 ± 1.0 TDAS ADD: 19.6 ± 1.7	MMSE, ADAS-j Cog, TDAS	NA	Intraoral dropping method (sweet, salty, sour, bitter) (RT) Subjective questionnaire on taste function	No difference between groups in the gustatory threshold Gustatory test score correlated with MMSE, ADAS-j Cog and TDAS scores *No difference between groups in subjective gustatory function*
Doorduijn et al. 2020	Patients: 52 (M: 30, W: 22; age: 69.6 ± 8.5) Controls: 40 (M: 18, W: 22; age: 62.5 ± 6.8)	MCI (NIA-AA criteria, 2011) Probable AD (NIA-AA criteria, 2011)	NA	MMSE MCI: 26^†^ (25–28)* MMSE probable AD: 24^†^ (21–26)*	MMSE	Memory, attention, executive function, language, visuospatial	Taste Strips Test (sweet, salty, sour, bitter) (ID) Taste intensity preference task Food preference computerized task	Overall gustatory function did not differ across groups *No difference between groups in taste intensity and food preference*

**List of abbreviations**: AD = Alzheimer’s Disease; ADAS-j Cog = Alzheimer’s Disease Assessment Scale-cognitive subscale Japanese version; aMCI = amnestic mild cognitive impairment; CDR = Clinical Dementia Rating Scale; CERAD = Consortium to Establish a Registry for Alzheimer’s Disease; DRS = Dementia Rating Scale; DSM = Diagnostic and Statistical Manual of Mental Disorders; DT = detection threshold; ID = identification; IMC = Information-Memory-Concentration test; M = men; MCI = Mild Cognitive Impairment; MMSE = Mini Mental State Examination; MRI = Magnetic Resonance Imaging; NA = not assessed; NIA-AA = National Institute on Aging-Alzheimer’s Association criteria; NIH = National Institute of Health; NINCDS-ADRDA = National Institute of Neurological and Communicative Diseases and Stroke/Alzheimer’s Disease and Related Disorders Association; NPI = Neuropsychiatric Inventory; RT = recognition threshold; TDAS = Touch Panel-type Dementia Assessment Scale; W = women. ^§^ = Validated tests: plain text; non validated tests: italics; ^†^ = median; * = Interquartile range

***Studies using a clinical diagnosis of AD.*** A cross-sectional study comparing AD patients to controls found neither significant between-group difference in sweet threshold, nor correlation between taste threshold and cognition scores in the AD group (Murphy et al., 1990). Similar results were reported in a subsequent study by the same authors (Murphy et al., 1999) including AD patients with different degrees of cognitive impairment (i.e., very mild, mild, moderate dementia) and age-matched healthy controls.

In a cross-sectional study, worse total and single taste quality scores were reported for both MCI and AD patients than healthy age- and sex-matched controls, but no difference was found when comparing MCI to AD groups (Steinbach et al., 2010).

A study examining taste detection (i.e., the threshold at which the subjects felt any taste) and recognition (i.e., the threshold at which the subjects felt a specific taste) thresholds in AD of different severity, reported (a) higher total values for both overall measures in patients than controls; (b) higher detection thresholds for sweet, salty and bitter taste, and higher recognition thresholds for sweet and sour taste when comparing AD to controls; and (c) cognitive dysfunction severity significantly associated to both total threshold values in the multivariate model (Sakai et al., 2016).

A cross-sectional study exploring gustatory dysfunction by means of chemical (i.e., FPD) and electrical (i.e., electrogustometry) methods found gustatory impairment to FPD, but not to electrogustometry, in AD versus controls (Ogawa et al., 2017).

A retrospective analysis of data from the National Health and Nutrition Examination Survey found inability to identify salty taste to be significantly associated to dementia but not MCI (Churnin et al., 2019).

A cross-sectional study reported poorer taste performance in AD patients with moderate cognitive impairment compared to age-matched controls (i.e., worse overall, bitter, and salty taste identification) and AD patients with milder levels of cognitive impairment (i.e., worse sweet taste identification) (Contri-Degiovanni et al., 2020).

In a whole-mouth procedure study, worse taste performance (i.e., higher total threshold values) was reported only in patients with dementia but not MCI when compared to age-matched controls (Kouzuki et al., 2020).

***Studies using a combined clinical and biomarkers diagnosis of AD***. A cross-sectional study including patients with amnestic MCI and AD dementia neither found a difference between patients and controls in gustatory threshold, nor a significant association between AD cerebrospinal biomarkers (i.e., Aβ 42 and phospho-tau 181 levels) and taste performance (Kouzuki et al., 2018).

A prospective cohort study exploring the association between gustatory function, cognitive domain involvement, and altered food preference in AD reported no difference in overall gustatory function between patients with MCI and AD. Because of this negative finding, the association between single cognitive domain dysfunction, AD cerebrospinal biomarkers and taste dysfunction was not explored, despite being originally planned (Doorduijn et al., 2020).

### Studies on Patients with Neurocognitive Disorders due to Parkinson’s Disease

Three studies examined gustatory dysfunction in patients with PD (Cecchini et al., 2019b; Masala et al., 2020; Nigam et al., 2021) (Table 3).

**Table 3:** Studies on patients with cognitive dysfunction due Parkinson’s disease (PD).

Ref.	Population	PD duration (years)	PD medication (LEDD)	Motor symptoms	Non-motor symptoms	Cognitive dysfunction severity	Cognitive testing		Gustatory testing (taste function assessed)	Results
							Screening	Tested domains		
Cecchini et al. 2019b	Patients: 50 (M: 29, W: 21; age: 68.1 ± 10.5) Controls: 50 (M: 23, W: 27; age: 67.5 ± 9.4)	PD-MCI: 5.8 ± 5.0 PD-NC: 5.2 ± 4.5	PD-MCI: 665 ± 513 mg PD-NC: 484 ± 313 mg	MDS-UPDRS III PD-MCI: 28.4 ± 16.0 MDS-UPDRS III PD-NC: 20.0 ± 13.4 H-Y PD-MCI: 1.9 ± 0.9 H-Y PD-NC: 1.5 ± 0.6	Depression (HAD) Apathy (AES-S)	MMSE PD-MCI: 26.5 ± 2.1 MMSE PD-NC: 29.0 ± 1.5 MoCA PD-MCI: 23.1 ± 1.8 MoCA PD-NC: 25.3 ± 1.3	MMSE, MoCA	Memory, attention, executive function, visuospatial, language	Whole Mouth Test (sweet, salty, sour, bitter) (ID) Taste Strips Test (sweet, salty, sour, bitter) (ID)	Sweet score measured with the Taste Strips Test was worse in PD than controls Global scores and sour were worse in PD-MCI + than PD-MCI Global scores, sour and salty identification were worse in PD patients with executive dysfunction compared to PD patients with normal cognition
Masala et al. 2020	Patients: 64 (M: 38, W: 26; age: 69.2 ± 10.1) Controls: 49 (age: 67.9 ± 9.6)	PD: 4.6 ± 3.7	PD: 320.5 ± 284.9 mg	MDS-UPDRS III PD: 23.9 ± 13.1 H-Y PD: 2.9 ± 4.4	Fatigue (PFS) Apathy (SAS)	MoCA PD-MCI+: 16.61 ± 3.86 MoCA PD-MCI-: 25.6 ± 3.6	MoCA	NR	Taste Strips Test (sweet, salty, sour, bitter) (ID)	Global and single taste quality scores were worse in PD than controls Global and single taste quality scores were worse in PD-MCI + than PD-MCI-^†^
Nigam et al. 2021	Patients: 44 iRBD (M: 37, W: 7; age: 68.4 ± 9.0); 19 PD (M: 11, W: 8; age: 72.2 ± 7.2) Controls: 29 (M: 22, W: 7; age: 63.9 ± 12.4)	PD: 7.8 ± 6.6	NR	MDS-UPDRS III PD: 13.2 ± 7.6	Sleepiness (ESS)	MoCA PD: 26.4 ± 3.2	MoCA	NR	Taste Strips Test (sweet, salty, sour, bitter) (ID)	iRBD and PD showed worse global and single taste quality scores than controls No difference between iRBD and PD No difference between PD-MCI + and PD-MCI-^†^

**List of abbreviations**: AES-S = apathy evaluation self-report scale; ESS = Epworth sleepiness score; HAD = Hamilton depression rating scale; H-Y = modified Hoehn and Yahr staging scale; iRBD = isolated REM sleep behavior disorder; LEDD = levodopa equivalent daily dose; M = men; MCI = mild cognitive impairment; MDS-UPDRS III = Movement Disorder Society Unified Parkinson’s disease rating scale part III; MMSE = Mini Mental State Examination; MoCA = Montreal Cognitive Assessment; NC = normal cognition; NR = not reported; PD = Parkinson’s disease; PFS = Parkinson’s Disease Fatigue Scale; SAS = Starkstein Apathy Scale; W = women. ^†^ = secondary analysis of original data requested to the corresponding Author of the study

In a cross-sectional, case-control study, PD patients were found to score lower than controls for sweet taste perception measured with the TST. The study also explored the association between PD-MCI, single cognitive domain involvement and taste function and found TST global scores and sour to be significantly worse in PD patients with than those without MCI, and executive impairment to be associated with worse TST global scores, sour and salty identification (Cecchini et al., 2019b).

Worse global and single taste scores were found in PD patients compared to controls in a study originally aimed to explore the correlation between gustatory dysfunction, other non-motor symptoms and weight in PD. Our secondary analysis of original data documented worse TST global score, sweet, salty, sour, and bitter identification in PD patients with than those without MCI (Masala et al., 2020).

A cross-sectional, case-control study including both prodromal PD patients (i.e., patients with isolated REM sleep behavior disorder [iRBD]) and patients with overt PD found worse overall TST and single taste scores in the iRBD and PD groups than controls, but no significant difference when comparing iRBD to PD. Our secondary analysis found no difference in gustatory measures when comparing PD patients with versus those without MCI (Nigam et al., 2021).

### Studies on Patients with Neurocognitive Disorders due to Other or Mixed Etiologies

Five studies explored gustatory dysfunction in patients with NCDs due to other or mixed etiologies (Schiffman et al., 1990; Lang et al., 2006; Brion et al., 2015; Naudin et al., 2015; Sakai et al., 2017) (Table 4).

**Table 4:** Studies on patients with cognitive dysfunction due to other/mixed etiologies

Ref.	Population	Diagnosis	Neuropsychiatric comorbidity	Cognitive dysfunction severity	Cognitive testing		Gustatory testing (taste function assessed)	Results^§^
					Screening	Tested domains		
Schiffman et al. 1990	Patients: 54 (age: 67.5 ± 9.1) Controls: 37 of whom 24 were age-matched (age: 77.5 ± 9.5)	Probable AD (NINCDS-ADRDA criteria, 1984): N 30 Possible AD (NINCDS-ADRDA criteria, 1984): N 8 Multi-infarctual or subcortical dementia: N 16	NA	MMSE probable AD: 16.2 ± 8.1 MMSE possible AD: 22.9 ± 6.7 MMSE other dementias: 20.5 ± 10.9	MMSE	NA	Staircase procedure (umami, bitter) (DT)	Umami DTs were higher in patients with dementia compared to age-matched controls
Lang et al. 2006	Patients: 52 (M: 26, W: 26; age: 71.9 ± 10.0) Controls: 52 (M: 26, W: 26; age: 71.2 ± 9.4)	AD (DSM-IV criteria): 24 of which 5 with an additional vascular component and 1 with PD Non-AD dementia: 28 (i.e., PD-D, LBD, FTD, hydrocephalus, MSA, CBD, MS)	NA	MMSE: 22.4 ± 4.8	MMSE	NA	Whole Mouth Test (sweet, salty, sour, bitter) (ID) Taste Strips Test (sweet, salty, sour, bitter) (ID)	AD patients performed worse than non-AD patients and controls for the Taste Strips and Whole-Mouth tests Parkinsonian patients (i.e., PD + AD, PD-D, LBD) performed worse on Whole-Mouth Test (sour) and Taste-Strips Test (salty) compared to other patients Significant moderate correlation between dementia severity and taste
Brion et al. 2015	Patients: 40 (M: 21, W: 19; age: 52.7 ± 8.7) Controls: 20 (M: 9, W: 11; age: 53.2 ± 5.3)	KS (DSM-IV criteria) AUD (DSM-IV criteria)	Depression (BDI) Anxiety (STAI) Alcohol consumption	NA	NA	NA	Taste Strip Test (sweet, salty, sour, bitter) (ID)	Taste total scores were worse for the patients compared to controls
Naudin et al. 2015	Patients: 40 (M: 11, W: 29; age: 69.0 ± 11.8) Controls: 24 (M: 9, W: 15; age: 67.4 ± 12.9)	MDD (DSM-IV criteria): 20 AD (McKhann criteria, 2011): 20	Depression (MADRS) Anhedonia (PAS, SAS)	MADRS MDD: 29.7 ± 7.7 MADRS AD: 8.6 ± 6.3 MMSE MDD: 24.9 ± 3.0 MMSE AD: 19.4 ± 3.1	MMSE	NA	Taste Identification Test (AFNOR) (sweet, salty, sour, bitter) (ID)	AD patients performed worse than MDD and controls in taste ID AD scored lower than controls for salty, sour, bitter, and sweet taste qualities
Sakai et al. 2017	Patients: 36 (M: 15, W: 21; age: 68.3 ± 7.3) Controls: 22 (M: 9, W: 13; age: 68.0 ± 8.0)	SD (Gorno Tempini et al., 2011 clinical criteria and MRI plus SPECT findings): 18 Probable AD (NINCDS–ADRDA criteria, 1984 and MRI plus SPECT findings): 18	Behavioral and psychiatric symptoms (NPI)	CDR SD 0.5: 14 patients CDR SD 1: 4 patients CDR AD 0.5: 11 patients CDR AD 1: 7 patients	MMSE	Memory, premorbid intellective ability, executive function	Filter Paper Discs Test (sweet, salty, sour, bitter) (DT + RT) Taste discrimination task Taste identification task	SD and AD showed higher DT and RT values than controls SD showed higher DT for sweet, salty, sour + higher RT for salty, sour, bitter than controls AD showed higher RT for salty and sour than controls *SD and AD showed impaired taste identification*

**List of abbreviations**: AUD = alcohol use disorder; AD = Alzheimer’s Disease; BDI = Beck Depression Inventory; CBD = cortico-basal degeneration; CDR = Clinical Dementia Rating Scale; DRS = Dementia Rating Scale; DSM = Diagnostic and Statistical Manual of Mental Disorders; DT = detection threshold; FTD = Fronto-Temporal dementia; ID = identification; KS = Korsakoff Syndrome; LBD = Lewy Body dementia; M = men; MADRS = Montgomery–Asberg Depression Rating Scale; MDD = Major Depressive Disorder; MMSE = Mini Mental State Examination; MS = multiple sclerosis; MSA = multi-system atrophy; NA = not assessed; NINCDS-ADRDA = National Institute of Neurological and Communicative Diseases and Stroke/Alzheimer’s Disease and Related Disorders Association; NPI = Neuropsychiatric Inventory; PAS = Physical Anhedonia Scale; PD-D: dementia associated to Parkinson’s Disease; RT = recognition threshold; SAS = Social Anhedonia Scale; SD = Semantic dementia; STAI = State and Trait Anxiety Inventory; W = women. ^§^ = Validated tests: plain text; non validated tests: italics

Increased thresholds for umami taste were found in a heterogeneous cohort including patients with dementia due to probable or possible AD and other etiologies (i.e., multi-infarct or subcortical dementia) compared to age-matched controls, but no significant correlation was found between taste and cognition scores for AD patients (Schiffman et al., 1990).

Patients with dementia due to different causes including AD, PD and other parkinsonisms, FTD, and other mixed conditions, performed worse than age-matched controls on two different taste identification tests, namely, the Whole Mouth Test (WMT) and TST in a pilot study. Patients with dementia and a parkinsonian syndrome showed worse scores for sour and salty taste on WMT and TST, respectively, than those without parkinsonism. A significant moderate correlation was found between the severity of dementia and taste impairment in the whole sample (Lang et al., 2006).

Worse taste identification in the TST was also reported in alcohol use disorder and Korsakoff syndrome, compared to age-matched controls (Brion et al., 2015).

A pilot cross-sectional study reported AD patients to perform worse in a taste identification test than age-matched patients with major depression disorder (MDD) and healthy controls, but no difference between MDD and controls. No significant correlations were found between the severity of cognitive or mood changes and taste function in MDD and AD patients (Naudin et al., 2015).

In a cross-sectional study including patients with AD and semantic dementia (Gorno-Tempini et al., 2011), the latter group showed significantly higher detection and recognition threshold total scores, higher thresholds for the detection of sweet and salty tastes, and for the recognition of salty, sour, and bitter tastes than controls (Sakai et al., 2017).

### Risk of Bias and Quality of the Studies

The NOS results showed that the mean overall score was 4.4 out of 9, which indicated that the methodological quality of the included studies was generally moderate. According to the NOS checklist, five studies were considered of low quality, 12 of moderate quality and two of high quality (Table 5).

**Table 5:** Risk of bias and study quality assessment for the included studies

Study	Selection	Comparability	Exposure	Total score
Murphy et al. 1990	***	-	-	3/9
Schiffman et al. 1990	*	-	*	2/9
Murphy et al. 1999	***	-	*	4/9
Churnin et al. 2019	**	*	**	5/9
Lang et al. 2006	*	**	*	4/9
Steinbach et al. 2010	****	**	*	7/9
Brion et al. 2015	***	**	*	6/9
Naudin et al. 2015	***	**	*	6/9
Sakai et al. 2016	***	-	*	4/9
Ogawa et al. 2017	**	-	*	3/9
Sakai et al. 2017	*	**	*	4/9
Kouzuki et al. 2018	*	-	*	2/9
Cecchini et al. 2019b	****	**	*	7/9
Contri-Degiovanni et al. 2020	**	-	*	3/9
Doorduijn et al. 2020	***	-	*	4/9
Kouzuki et al. 2020	***	-	*	4/9
Masala et al. 2020	****	-	*	5/9
Nigam et al. 2021	***	**	*	6/9

### Meta-analyses

Thirty-five meta-analyses were carried out on taste performance (i.e., threshold, identification) in patients with NCDs due to AD or PD. Global, sweet, salty, sour, and bitter scores were explored as outcome measures. Different taste features (i.e., threshold, identification) were separately meta-analyzed. As the number of studies was low, moderator analysis and visual inspection of funnel plots for publication bias could not be performed (Borenstein, 2009b). Detailed information on the specific effect sizes for each of the included studies and results of the meta-analyses are displayed graphically in the forest plots (Fig. S1-S7) and summarized in Table 6, respectively.

**Table 6:** Results of the meta-analyses

**Outcome**	K	**N**	Random-effect model results				Heterogeneity				
			**MD**	**[95% CI]**	**Z**	***p***	***Q***	**df**	***p***	**τ** ^**2**^	**I**^**2**^ **(%)**
**DT**											
**Alzheimer’s disease (AD)**											
*AD vs controls, FPD test (positive MD values indicate worse performance for patients than controls)*											
FPD score	2	97	3.28	[1.02, 5.53]	2.85	**0.004**	9.35	1	0.002	2.36	89
Sweet	2	97	0.76	[0.43, 1.09]	4.50	**< 0.00001**	0.79	1	0.37	0.00	0
Salty	2	97	0.79	[0.20, 1.37]	2.63	**0.009**	4.88	1	0.03	0.14	79
Sour	2	97	0.75	[-0.14, 1.63]	1.66	0.10	8.96	1	0.003	0.36	89
Bitter	2	97	0.87	[0.29, 1.46]	2.93	**0.003**	2.67	1	0.10	0.11	63
**RT**											
*AD vs controls, FPD test (positive MD values indicate worse performance for patients than controls)*											
FPD score	2	97	4.65	[2.80, 6.50]	4.92	**< 0.00001**	2.92	1	0.09	1.19	66
Sweet	2	97	0.77	[0.35, 1.20]	3.59	**0.0003**	0.20	1	0.65	0.00	0
Salty	2	97	1.25	[0.47, 2.03]	3.16	**0.002**	2.26	1	0.13	0.18	56
Sour	2	97	1.30	[0.78, 1.82]	4.91	**< 0.00001**	0.00	1	1.00	0.00	0
Bitter	2	97	1.26	[0.38, 2.14]	2.81	**0.005**	3.86	1	0.05	0.30	74
**ID**											
**Alzheimer’s disease (AD)**											
*MCI vs controls, TST (negative MD values indicate worse performance for patients than controls)*											
TST score	3	187	-0.86	[-2.80, 1.08]	0.87	0.39	26.82	2	< 0.00001	2.62	93
Sweet	3	187	0.09	[-0.37, 0.55]	0.37	0.71	10.57	2	0.005	0.13	81
Salty	3	187	-0.57	[-1.52, 0.38]	1.17	0.24	29.96	2	< 0.00001	0.65	93
Sour	3	187	-0.04	[-0.49, 0.41]	0.17	0.86	13.78	2	0.001	0.13	85
Bitter	3	187	-0.27	[-0.71, 0.16]	1.23	0.22	7.64	2	0.02	0.11	74
*AD vs controls, TST (negative MD values indicate worse performance for patients than controls)*											
TST score	3	182	-2.26	[-4.56, 0.03]	1.93	**0.05**	30.76	2	< 0.00001	3.76	93
Sweet	3	182	-0.38	[-0.87, 0.10]	1.56	0.12	8.54	2	0.01	0.14	77
Salty	3	182	-0.70	[-1.92, 0.52]	1.13	0.26	44.42	2	< 0.00001	1.09	95
Sour	3	182	-0.56	[-0.86, -0.27]	3.72	**0.0002**	4.82	2	0.09	0.04	58
Bitter	3	182	-0.61	[-1.28, 0.05]	1.81	0.07	16.34	2	0.0003	0.29	88
*AD vs MCI, TST (negative MD values indicate worse performance for AD than MCI)*											
TST score	3	171	-1.22	[-1.47, -0.97]	9.59	**< 0.00001**	1.59	2	0.45	0.00	0
Sweet	3	171	-0.41	[-0.51, -0.30]	7.47	**< 0.00001**	1.46	2	0.48	0.00	0
Salty	3	171	0.05	[-0.16, 0.26]	0.46	0.65	2.26	2	0.32	0.01	11
Sour	3	171	-0.55	[-0.92, -0.19]	2.97	**0.003**	6.96	2	0.03	0.07	71
Bitter	3	171	-0.25	[-0.56, 0.06]	1.56	0.12	4.03	2	0.13	0.04	50
**Parkinson’s disease (PD)**											
*PD-MCI + vs controls, TST (negative MD values indicate worse performance for patients than controls)*											
TST score	3	193	-3.79	[-7.52, -0.05]	1.99	**0.05**	27.87	2	< 0.00001	9.88	93
Sweet	3	193	-1.06	[-1.75, -0.37]	3.00	**0.003**	9.08	2	0.01	0.28	78
Salty	3	193	-1.19	[-2.12, -0.27]	2.54	**0.01**	14.71	2	0.0006	0.56	86
Sour	3	193	-0.94	[-1.54, -0.35]	3.10	**0.002**	6.03	2	0.05	0.18	67
Bitter	3	193	-0.89	[-2.05, 0.27]	1.50	0.13	18.27	2	0.0001	0.91	89
*PD-MCI + vs PD-MCI-, TST (negative MD values indicate worse performance for PD-MCI + than PD-MCI-)*											
TST score	3	128	-1.74	[-2.75, -0.72]	3.35	**0.0008**	0.38	2	0.83	0.00	0
Sweet	3	128	-0.40	[-0.76, -0.04]	2.15	**0.03**	1.67	2	0.43	0.00	0
Salty	3	128	-0.47	[-0.84, -0.10]	2.51	**0.01**	1.27	2	0.53	0.00	0
Sour	3	128	-0.55	[-0.95, -0.15]	2.70	**0.007**	2.10	2	0.35	0.01	5
Bitter	3	128	-0.29	[-0.66, 0.08]	1.54	0.12	0.49	2	0.78	0.00	0

**List of abbreviations**: CI = confidence interval; DT = detection threshold; FPD = Filter Paper Discs test; ID = identification; K = number of studies; MCI = mild cognitive impairment; MD = mean difference; N = number of participants; PD-MCI+/- = Parkinson’s disease with/without mild cognitive impairment; RT = recognition threshold; TST = Taste Strips Test. *P* values ≤ 0.05 are reported in bold type

### Taste Function in Neurocognitive Disorders due to Alzheimer’s Disease

Separate meta-analyses were conducted for studies assessing taste threshold and identification. Studies assessing detection (i.e., the threshold at which the subjects felt any taste) and recognition (i.e., the threshold at which the subjects felt a specific taste) threshold and those including patients with MCI and dementia were separately meta-analyzed, too.

***Studies assessing detection threshold with the FPD test.*** The meta-analysis on the association between NCDs due to AD and detection threshold measured with the FPD test included 97 subjects. Controls showed better global (MD = 3.28; 95% CI: 1.02, 5.53; *p* = 0.004), sweet (MD = 0.76; 95% CI: 0.43, 1.09; *p* < 0.0001), salty (MD = 0.79; 95% CI: 0.20, 1.37; *p* = 0.009) and bitter (MD = 0.87; 95% CI: 0.29, 1.46; *p* = 0.003) scores compared to AD. Sour scores (MD = 0.75; 95% CI: -0.14, 1.63; *p* = 0.10) did not differ between groups. Heterogeneity was significant for global (*Q* [1] = 9.35, *p* = 0.002; τ^2^ = 2.36; I^2^ = 89%), salty (*Q* [1] = 4.88, *p* = 0.03; τ^2^ = 0.14; I^2^ = 79%) and sour (*Q* [1] = 8.96, *p* = 0.003; τ^2^ = 0.36; I^2^ = 89%) scores and not significant for sweet and bitter scores (Fig. S1).

***Studies assessing recognition threshold with the FPD test.*** The meta-analysis on the association between NCDs due to AD and recognition threshold measured with the FPD test included 97 subjects. Controls showed better global (MD = 4.65; 95% CI: 2.80, 6.50; *p* < 0.00001), sweet (MD = 0.77; 95% CI: 0.35, 1.20; *p* = 0.0003), salty (MD = 1.25; 95% CI: 0.47, 2.03; *p* = 0.002), sour (MD = 1.30; 95% CI: 0.78, 1.82; *p* < 0.00001) and bitter (MD = 1.26; 95% CI: 0.38, 2.14; *p* = 0.005) scores compared to AD. Heterogeneity was borderline for significance for bitter (*Q* [1] = 3.86, *p* = 0.05; τ^2^ = 0.30; I^2^ = 74%) score and not significant for global, sweet, salty and sour scores (Fig. S2).

***Studies assessing taste identification with the TST.*** Separate meta-analyses on the association between NCDs due to AD and taste identification measured with the TST were performed for patients with mild (i.e., MCI) and major (i.e., dementia) NCDs. Effect sizes for global (MD = -0.86; 95% CI: -2.80, 1.08; *p* = 0.39), sweet (MD = 0.09; 95% CI: -0.37, 0.55; *p* = 0.71), salty (MD = -0.57; 95% CI: -1.52, 0.38; *p* = 0.24), sour (MD = -0.04; 95% CI: -0.49, 0.41; *p* = 0.86), and bitter (MD = -0.27; 95% CI: -0.71, 0.16; *p* = 0.22) scores did not differ significantly between MCI and controls. Heterogeneity was significant for global (*Q* [2] = 26.82, *p* < 0.00001; τ^2^ = 2.62; I^2^ = 93%), sweet (*Q* [2] = 10.57, *p* = 0.005; τ^2^ = 0.13; I^2^ = 81%), salty (*Q* [2] = 29.96, *p* < 0.00001; τ^2^ = 0.65; I^2^ = 93%), sour (*Q* [2] = 13.78, *p* = 0.001; τ^2^ = 0.13; I^2^ = 85%) and bitter (*Q* [2] = 7.64, *p* = 0.02; τ^2^ = 0.11; I^2^ = 74%) scores (Fig. S3).

AD patients showed worse global (MD = -2.26; 95% CI: -4.56, 0.03; *p* = 0.05) and sour (MD = -0.56; 95% CI: -0.86, -0.27; *p* = 0.0002) scores compared to controls. Sweet (MD = -0.38; 95% CI: -0.87, 0.10; *p* = 0.12), salty (MD = -0.70; 95% CI: -1.92, 0.52; *p* = 0.26) and bitter (MD = -0.61; 95% CI: -1.28, 0.05; *p* = 0.07) scores did not differ between groups. Heterogeneity was significant for global (*Q* [2] = 30.76, *p* < 0.00001; τ^2^ = 3.76; I^2^ = 93%), sweet (*Q* [2] = 8.54, *p* = 0.01; τ^2^ = 0.14; I^2^ = 77%), salty (*Q* [2] = 44.42, *p* < 0.00001; τ^2^ = 1.09; I^2^ = 95%) and bitter (*Q* [2] = 16.34, *p* = 0.0003; τ^2^ = 0.29; I^2^ = 88%) scores, and not significant for sour score (Fig. S4).

AD showed worse global (MD = -1.22; 95% CI: -1.47, -0.97; *p* < 0.00001), sweet (MD = -0.41; 95% CI: -0.51, -0.30; *p* < 0.00001) and sour (MD = -0.55; 95% CI: -0.92, -0.19; *p* = 0.003) scores compared to MCI patients. Salty (MD = 0.05; 95% CI: -0.16, 0.26; *p* = 0.65) and bitter (MD = -0.25; 95% CI: -0.56, 0.06; *p* = 0.12) scores did not differ between groups. Heterogeneity was significant for sour (*Q* [2] = 6.96, *p* = 0.03; τ^2^ = 0.07; I^2^ = 71%) score and not significant for global, sweet, salty and bitter scores (Fig. S5).

### Taste Function in Neurocognitive Disorders due to Parkinson’s Disease

The meta-analyses on the association between NCDs due to PD and taste function included 193 subjects. Since the studies by Masala et al. (2020) and Nigam et al. (2021) did not provide information on the number of PD patients with cognitive impairment, exact data on cognitive measures were requested from the authors and the presence (PD-MCI+; N = 65 patients) and absence of PD-MCI (PD-MCI-; n = 63 patients) was defined according to Movement Disorder Society (MDS) level I criteria (Litvan et al., 2012) and validated cut-offs for Montreal Cognitive Assessment (Federico et al., 2015; Dujardin et al., 2016).

PD-MCI + showed worse global (MD = -3.79; 95% CI: -7.52, -0.05; *p* = 0.05), sweet (MD = -1.06; 95% CI: -1.75, -0.37; *p* = 0.003), salty (MD = -1.19; 95% CI: -2.12, -0.27; *p* = 0.01), and sour (MD = − 0.94; 95% CI: -1.54, -0.35; *p* = 0.002) scores compared to controls, while bitter score (MD = -0.89; 95% CI: -2.05, 0.27; *p* = 0.13) did not differ between groups. Heterogeneity was significant for global (*Q* [2] = 27.87, *p* < 0.00001; τ^2^ = 9.88; I^2^ = 93%), sweet (*Q* [2] = 9.08, *p* = 0.01; τ^2^ = 0.28; I^2^ = 78%), salty (*Q* [2] = 14.71, *p* = 0.0006; τ^2^ = 0.56; I^2^ = 86%) and bitter (*Q* [2] = 18.27, *p* = 0.0001; τ^2^ = 0.91; I^2^ = 89%) scores, and borderline for significance for sour (*Q* [2] = 6.03, *p* = 0.05; τ^2^ = 0.18; I^2^ = 67%) score (Fig. S6).

PD-MCI + showed worse global (MD = -1.74; 95% CI: -2.75, -0.72; p = 0.0008), sweet (MD = -0.40; 95% CI: -0.76, -0.04; p = 0.03), salty (MD = -0.47; 95% CI: -0.84, -0.10; p = 0.01) and sour (MD = -0.55; 95% CI: -0.95, -0.15; p = 0.007) scores compared to PD-MCI-, with no between-groups difference for bitter score (MD = 0.12; 95% CI: -0.66, 0.08; *p* = 0.12). The heterogeneity was not significant for all the above-mentioned measures (*Q* values ranging from 0.38 to 2.10; τ^2^ values ranging from 0.00 to 0.01; I^2^ values ranging from 0 to 5%) (Fig. S7).

### Sensitivity Analyses

Sensitivity analyses were performed for meta-analyses where at least three studied were included, and either heterogeneity was borderline for significance, statistically significant, or effect size was not significant. Detailed results are displayed in Table S3.

***NCDs due to AD.*** The overall effect size for sweet and salty scores became significant and heterogeneity became not significant after removing Steinbach et al. (2010) and Doorduijn et al. (2020), respectively, in the meta-analyses comparing MCI patients to controls. In the meta-analyses comparing AD patients to controls, after removing the studies by Steinbach et al. (2010), and Doorduijn et al. (2020), the effect sizes for all the outcomes became significant and heterogeneity became not significant. The effect sizes for sour and bitter scores became significant and heterogeneity became not significant after removing Doorduijn et al. (2020) in the meta-analyses comparing AD to MCI patients.

***NCDs due to PD.*** After removing Cecchini et al. (2019), the effect size for bitter score became significant and heterogeneity became not significant, in the meta-analyses comparing PD-MCI + patients and controls. Including or excluding other studies did not change the overall effect sizes for the other outcomes of interest but resulted in a reduction of heterogeneity that became not significant.

## Discussion

The main findings of this systematic review and meta-analysis of studies evaluating gustatory function in NCDs of different etiologies were as follows: (a) despite some discrepancy across studies, patients with NCDs showed overall worse gustatory function than cognitively intact individuals, (b) taste dysfunction was differentially associated with the severity of cognitive deficits in AD-related NCDs, (c) different gustatory features and taste qualities were differentially impaired in NCDs, and (d) gustatory dysfunction in NCDs did not differ according to etiology. We will discuss each of these findings in more details below.

### The Association Between Neurocognitive Disorders and Gustatory Dysfunction

Our systematic review documented conflicting findings on gustatory function in AD and related MCI, with six studies reporting a taste deficit in patients compared to controls (Steinback et al., 2010; Sakai et al., 2017; Ogawa et al., 2017; Churnin et al., 2019; Contri-Degiovanni et al., 2020; Kouzuki et al., 2020), and four reports yielding negative findings (Murphy et al., 1990, 1999; Kouzuki et al., 2018; Doorduijn et al., 2020). The meta-analysis showed worse taste detection threshold and identification in AD patients.

Two out of three studies converged in showing worse gustatory function in PD patients with MCI than cognitively intact PD patients and controls (Cecchini et al., 2019b; Masala et al., 2020). Only one study failed to report significant difference between PD patients with and without MCI (Nigam et al., 2021). The meta-analysis showed worse gustatory function in PD patients versus controls, and PD with MCI versus those without MCI.

Five studies on NCDs due to mixed etiologies, including dementia related to PD and parkinsonism, FTD, VaD, and semantic dementia, alcohol use disorder and Korsakoff syndrome, showed worse taste function than controls (Schiffman et al., 1990; Lang et al., 2006; Brion et al., 2015; Naudin et al., 2015; Sakai et al., 2017), but a meta-analysis was not feasible because of the heterogeneity of etiologies.

Taken together these findings converge and indicate taste function as a potential non-invasive biomarker of NCDs. These results also expand the knowledge on chemosensory alterations in NCDs by documenting the involvement of the gustatory system in addition to the olfactory one (Doty & Hawkes, 2019).

### Neurocognitive Disorder Severity May Be Associated to Taste Function

Only a few studies compared patients with AD-related MCI and dementia, with five of them showing similar gustatory involvement (Murphy et al., 1990, 1999; Steinback et al., 2010; Kouzuki et al., 2018; Doorduijn et al., 2020), while three reports showed worse gustatory performance in AD than MCI or mild AD (Churnin et al., 2019; Contri-Degiovanni et al., 2020; Kouzuki et al., 2020). The meta-analysis showed similar TST score in MCI than controls and significantly worse TST in AD compared to MCI.

Studies on PD did not allow a comparison between patients with dementia versus MCI because of the absence of studies recruiting both populations.

The use of whole mouth versus regional tests, with the former being not sensitive enough to detect subtle gustatory alterations (Doty, 2018), and the type of stimuli, with only one study applying electrogustometry (Ogawa et al., 2017), may have influenced the results of the included studies.

These findings indicate that overall gustatory dysfunction might be related to the severity of cognitive impairment, but additional data are required. Moreover, cognitive impairment may negatively impact the performance of psychophysical gustatory tests in patients with dementia. Because of this limitation, gustatory event related potentials or functional magnetic resonance imaging might offer more robust support to our results, but few specialized research centers apply these methods to taste assessment (Kobal, 1985; Hummel et al., 2007). Finally, taste and smell could be impaired by physiological ageing (Fukunaga et al., 2005) underlying age-related anorexia (Di Francesco et al., 2007), and this phenomenon could contribute to gustatory findings in NCDs that are typically age-related conditions.

### Neurocognitive Disorder is Associated to Different Gustatory Features

Taste detection or recognition threshold, identification, and intensity offer complementary information on gustatory function. The included studies explored identification (Lang et al., 2006; Steinbach et al., 2010; Brion et al., 2015; Naudin et al., 2015; Cecchini et al., 2019b; Churnin et al., 2019; Contri Degiovanni et al., 2020; Doorduijn et al., 2020; Masala et al., 2020; Nigam et al., 2021) or threshold (Murphy et al., 1990, 1999; Schiffman et al., 1990; Sakai et al., 2016, 2017; Ogawa et al., 2017; Kouzuki et al., 2018, 2020), but none assessed intensity.

Gustatory threshold and identification were reported to be worse in patients with dementia due to different etiologies in three studies (Sakai et al., 2016; Ogawa et al., 2017; Kouzuki et al., 2020) and similar to controls in two reports (Ogawa et al., 2017; Kouzuki et al., 2018). The meta-analyses showed overall worse gustatory detection and recognition thresholds in AD-dementia compared to controls. Two studies showed higher gustatory thresholds in dementia due to other or mixed etiologies compared to controls (Schiffman et al., 1990; Sakai et al., 2017).

Patients with MCI due to AD showed similar threshold than controls in two studies (Kouzuki et al., 2018, 2020), while we found no reports of threshold in patients with PD-MCI. The very low number of reports prevented further analyses, suggesting the need for future studies on this topic.

Identification, despite being assessed with different tools, was found to be impaired in most studies on patients with AD-dementia (Steinbach et al., 2010; Churnin et al., 2019; Contri-Degiovanni et al., 2020), a finding confirmed in the meta-analysis, and in three studies on dementia due to other or mixed aetiologies (Lang et al., 2006; Brion et al., 2015; Naudin et al., 2015). Only one study included patients with dementia related to parkinsonism, but the heterogeneity of the involved patients (i.e., PD, LBD, PD + AD) prevented further analyses (Lang et al., 2006).

Overall identification was reported to be abnormal in PD-MCI, in the qualitative and quantitative syntheses of three studies comparing PD patients with versus those without MCI (Cecchini et al., 2019b; Masala et al., 2020; Nigam et al., 2021).

To summarize, gustatory features appear to be differentially associated to NCD severity, with threshold being affected in major NCDs, and identification both in minor and major NCDs. Data on olfaction suggest that identification may rely on higher cognitive demand than detection, which is considered a low-level perceptual process (Hedner et al., 2010). Executive function and semantic memory, which are more severely affected in major versus minor NCDs, have been suggested to impact on olfactory identification (Dulay et al., 2008). The same reasoning may apply to gustatory identification, which may be more impaired with disease progression. Further studies should explore whether taste identification might be a biomarker of NCD severity (Sakai et al., 2016).

### Single Taste Qualities Identification in Neurocognitive Disorder

Identification of single taste qualities was reported either as spared (Doorduijn et al., 2020) or impaired (Lang et al., 2006; Steinbach et al., 2010; Naudin et al., 2015; Contri-Degiovanni et al., 2020) in patients with NCDs due to AD. Sweet (Steinbach et al., 2010; Naudin et al., 2015; Contri-Degiovanni et al., 2020), salty (Steinbach et al., 2010; Lang et al., 2006; Naudin et al., 2015; Contri-Degiovanni et al., 2020), sour (Lang et al., 2006; Steinbach et al., 2010; Naudin et al., 2015) and bitter identification (Lang et al., 2006; Steinbach et al., 2010; Naudin et al., 2015; Contri-Degiovanni et al., 2020) were abnormal in most of the included studies. Umami identification was assessed only in one study, with no difference between MCI, AD-dementia, and controls (Contri-Degiovanni et al., 2020). The meta-analysis showed worse sour identification in dementia compared to controls and worse sweet and sour identification in dementia versus MCI in AD.

Data appear to be robust in PD-MCI, with qualitative and quantitative syntheses showing altered sweet, salty, and sour identification but normal bitter identification when comparing PD patients with MCI to those without MCI (Cecchini et al., 2019b; Masala et al., 2020; Nigam et al., 2021) and to controls (Cecchini et al., 2019b; Masala et al., 2020; Nigam et al., 2021).

The differential degree of decline in each taste quality identification may reflect distinct biological significances of each taste. Sweet and salty tastes indicate high nutritional and mineral contents, respectively, and therefore are important to detect the most important sources of nutrients. Hence, abnormal sweet and salty identification may lead patients to shift towards unhealthy diet (Sergi et al., 2017), increasing the odds of obesity, metabolic and cardiovascular diseases (Imoscopi et al., 2012). On the other hand, sour and bitter tastes identify the presence of rotten and toxic foods, respectively (Chandrashekar et al., 2006; Reed et al., 2010) and preservation of bitter identification in patients with NCDs may reflect the evolutionary role and protective value of this taste to avoid ingesting dangerous foods (Wooding et al., 2021).

### Neurocognitive Disorder Etiologies Seem Not to Be Associated to Taste Dysfunction

We found no difference between distinct NCD etiologies, both overall and when considering single taste qualities. Only few studies explored the relationship between neuropathological biomarkers of NCDs (AD biomarkers, in particular) and gustatory alterations, and none of them found significant associations (Kouzuki et al., 2018; Doorduijn et al., 2020). Sweet, salty, sour, bitter, and umami detection and identification were reported to be affected irrespective of the etiological subtype of NCDs. Taken together, these findings suggest that gustatory dysfunction may act as a cross-disease chemosensory biomarker of NCDs, offering complementary information on cognitive alterations, especially when coupled with clinically established biomarkers, but further studies with larger samples are needed to confirm this hypothesis.

### The Anatomy of Gustatory Dysfunction in Neurocognitive Disorders

The central component of gustatory processing explains why NCDs may be associated to gustatory dysfunction. Brain regions that are involved in taste are also affected by NCDs (Sewards, 2004; Lang et al., 2006; Gasquoine, 2014; Doty & Hawkes, 2019). Amygdala, insula, orbitofrontal cortex, and thalamus are key brain regions both for gustatory and cognitive processing. Volume changes in the medial temporal lobe structures including amygdala, hippocampus, entorhinal cortex, and parahippocampal gyrus, have been reported by neuroimaging studies of early AD (Thangavel et al., 2008; Poulin et al., 2011). Medial temporal lobe atrophy is also common in FTD, with the amygdala being among the earliest structures to be affected especially in the genetic cases (Whitwell et al., 2012). The anterior insula is implicated in gustatory identification processes and the posterior insula contributes to oral somatic sensation (Veldhuizen et al., 2011; Nakamura et al., 2013; Rolls, 2016). Higher levels of insular atrophy have been documented in more advanced AD and FTD stages (Moon et al., 2014; Sakai et al., 2017). Insula and frontal operculum have also been regarded as key brain regions for gustatory detection and recognition threshold (Ogawa et al., 1994), features that were found to be significantly impaired in patients with semantic dementia, an FTD variant (Sakai et al., 2017). Functional alterations of the orbitofrontal cortex have been reported in patients with MCI due to AD (Schroeter et al., 2009). Functional and structural alterations of the insula and prefrontal cortex have been reported in PD patients with versus those without MCI (Mihaescu et al., 2018). Volume loss and altered functional connectivity of the thalamus, a key region involved in the modulation of cognition, were also documented in PD patients with MCI compared to cognitively intact ones (Li et al., 2020). Taken together, these findings support the view of shared anatomical brain regions underlying gustatory dysfunction and NCDs of distinct etiologies.

### The Relationship Between Olfactory and Gustatory Dysfunction in Neurocognitive Disorders

Olfactory and gustatory abnormalities have been reported in NCDs (Doty & Hawkes, 2019), but their relationship is still unclear. On one hand, olfactory and gustatory alterations have been proposed to be independent in the light of separate anatomical pathways of the first and second order neurons (Rolls, 2019). On the other side, mutual chemosensory interactions have been documented, with long-term olfactory loss leading to subtle decrease in gustatory identification (Landis et al., 2010). The amygdala, insula, and in particular the multimodal orbitofrontal cortex, are common brain sites where mutual modulation between olfactory, gustatory, and other sensory information takes place (Dalton et al., 2000; Shepherd 2006). Studies on NCDs patients including the assessment of both chemosensory functions, separately and by means of validated tests, may offer further information on this topic. Twelve out of the 18 included studies assessed both chemosensory functions (Murphy et al., 1990, 1999; Schiffman et al., 1990; Lang et al., 2006; Steinbach et al., 2010; Brion et al., 2015; Kouzuki et al., 2018; Cecchini et al., 2019b; Churnin et al., 2019; Doorduijn et al., 2020; Masala et al., 2020; Nigam et al., 2021), with conflicting results (Table S4). Most studies found simultaneously disrupted gustatory and olfactory abilities in patients with minor and major NCDs due to different etiologies (Schiffman et al., 1990; Lang et al., 2006; Steinbach et al., 2010; Brion et al., 2015; Cecchini et al., 2019b; Churnin et al., 2019; Masala et al., 2020; Nigam et al., 2021). Gustatory abilities were reported to be spared despite altered olfaction in a lower number of studies (Murphy et al., 1990, 1999; Kouzuki et al., 2018; Doorduijn et al., 2020). Moreover, one study found no correlation between olfaction and taste scores (Cecchini et al., 2019b). Further studies assessing chemoreception in NCDs are needed to explore if the combination of olfactory and gustatory scores might better stratify patients with NCDs.

### Strengths and Limitations

The strengths of this paper are (a) the use of a broad search strategy and three databases to reduce the risk of missing relevant papers, (b) the quantitative analysis to better explore the association between gustatory and cognitive dysfunctions, (c) the inclusion of studies assessing gustatory function with validated and standardized measures, and (d) the selection of studies providing objective data on cognitive dysfunction.

Our review has also some limitations. The small number of studies (i.e., 2–3) for each gustatory feature and NCD condition are likely to reflect imprecise estimates of effects and reducing the statistical power of the meta-analysis, especially for threshold, and precluded moderator analysis on variables that are known to influence gustatory function (e.g., age, medications, smoking status, alcohol consumption) (Frank et al., 1992; Sergi et al., 2017). Moreover, the small number of studies and the small sample sizes of the single studies may have further contributed to decrease the statistical power of the quantitative analysis, by introducing additional heterogeneity (Borenstein, 2009b; Hedges & Pigott, 2001). These methodological limitations, together, make the inference about heterogeneity and the comparison across groups difficult. Therefore, the results of the meta-analysis should be considered a descriptive summary tool, which needs to be interpreted cautiously. Included studies used different gustatory evaluation tools, further increasing the heterogeneity of results. Also, we were not able to perform direct comparisons between NCDs due to different etiologies, for example, AD, PD, FTD, and VaD. The effect of concomitant olfactory dysfunction could not be completely ruled out, as discussed above. Another limitation is the retrospective definition of PD-MCI according to level I MDS criteria for two out of three of the included studies on PD, which prevented us to better explore the role of single cognitive domain dysfunction on taste abnormalities (Cecchini et al., 2019b; Wallace et al., 2022).

## Conclusions and Future Directions

To conclude, our findings indicate that taste abnormalities are common in several NCDs due to different etiologies, suggesting that gustatory dysfunction may represent a potential cross-disease chemosensory biomarker of NCD. We also found that taste dysfunction was differentially associated with the severity of cognitive deficits and that different gustatory features and taste qualities could be differentially impaired in NCDs. Despite the methodological limitations discussed above, these findings might be of interest in clinical practice. Several studies attempted to identify highly specific biomarkers for NCDs, but yielded inconclusive results (Delgado-Alvarado et al., 2016; Huang et al., 2022). Sensitive biomarkers should accurately predict the evolution of NCDs that are characterized by proteinopathy, neurodegeneration, and changes in neurotransmission. Notwithstanding this complex picture, most studies focused on single biomarkers tested on small samples of patients, limiting their potential translation into clinical practice. Combined panels of biomarkers offering complementary information may improve diagnostic and prognostic accuracy (Vincent et al., 2020). The overlap between brain areas involved in taste and cognition support the view that gustatory testing might be used as NCD biomarker. Whether gustatory alterations may be used as screening tool for NCDs in high-risk populations, or to predict NCDs progression, requires further studies, which should use sensitive validated gustatory measures. In addition, we point out that new studies with larger samples, and assessing both olfaction and taste are needed to understand if the pattern of abnormalities in the two senses might better stratify patients with NCDs. The role of the combination of taste changes with clinical, biological samples, and imaging biomarkers should also be explored.

### Electronic Supplementary Material

Below is the link to the electronic supplementary material.

Supplementary Material 1

Supplementary Material 2

Supplementary Material 3

Supplementary Material 4

Supplementary Material 5

Supplementary Material 6

Supplementary Material 7

Supplementary Material 8

## Data Availability

Analyses were conducted using Review Manager (RevMan version 5.4, The Cochrane Collaboration).
