# Supplementary figures and images for: The Association Between Neurocognitive Disorders and Gustatory Dysfunction: A Systematic Review and Meta-Analysis
Source: Neuropsychol Rev. 2023 Feb 20;34(1):192–213. doi: 10.1007/s11065-023-09578-3 (PMC10920407; doi:10.1007/s11065-023-09578-3)

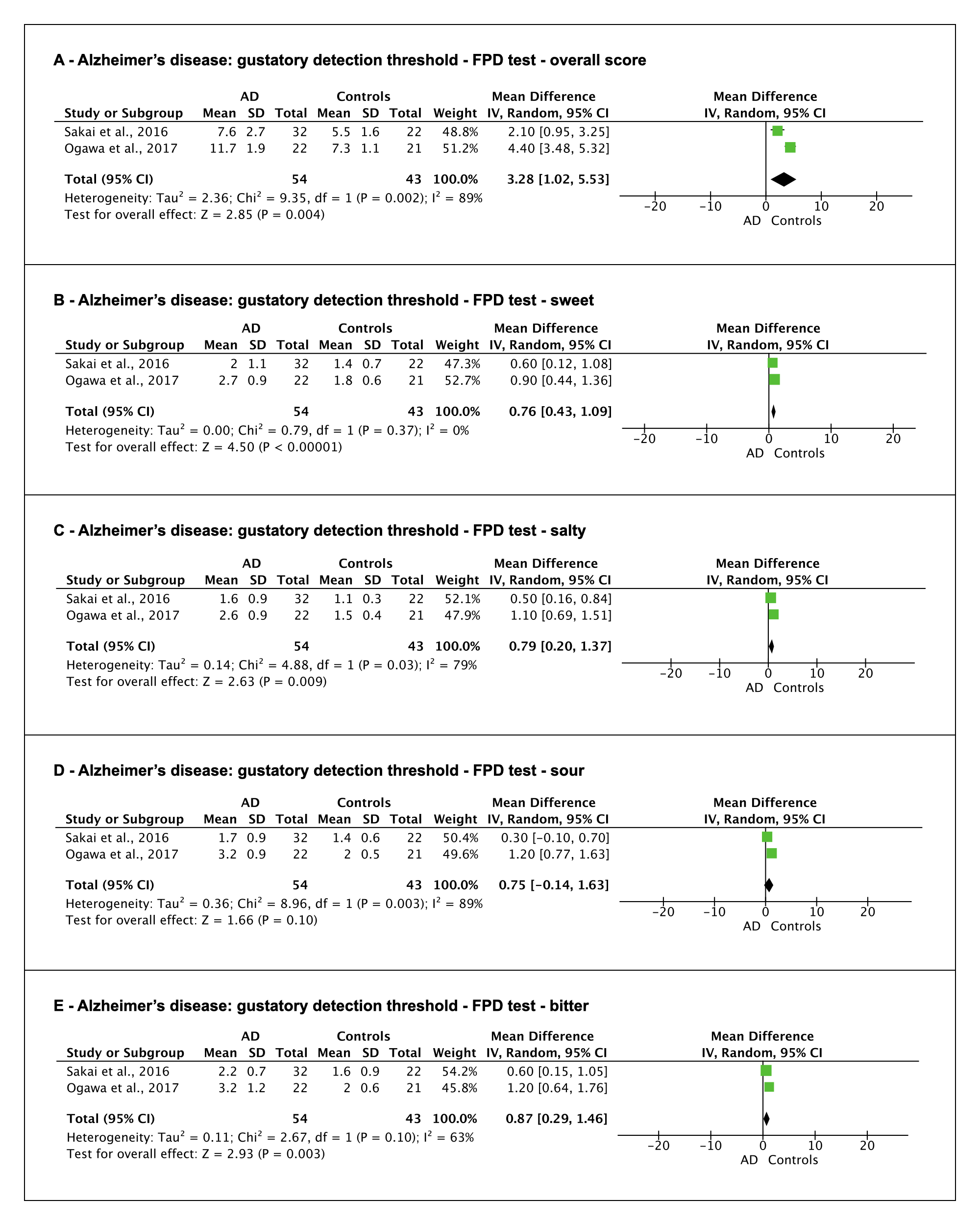

Supplement: Supplementary file 1 — Supplementary Material 1 [file 11065_2023_9578_MOESM1_ESM.jpeg]

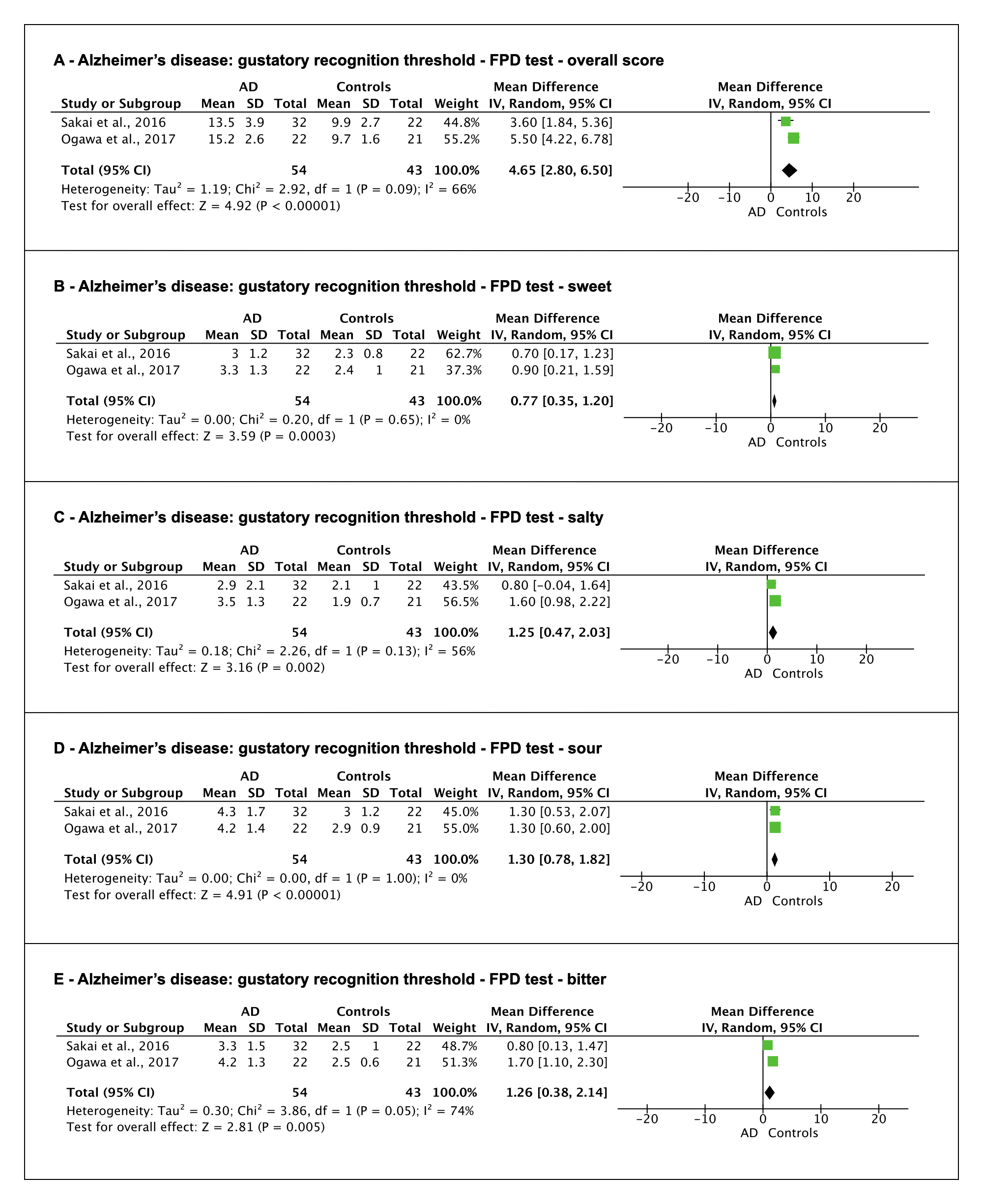

Supplement: Supplementary file 2 — Supplementary Material 2 [file 11065_2023_9578_MOESM2_ESM.jpeg]

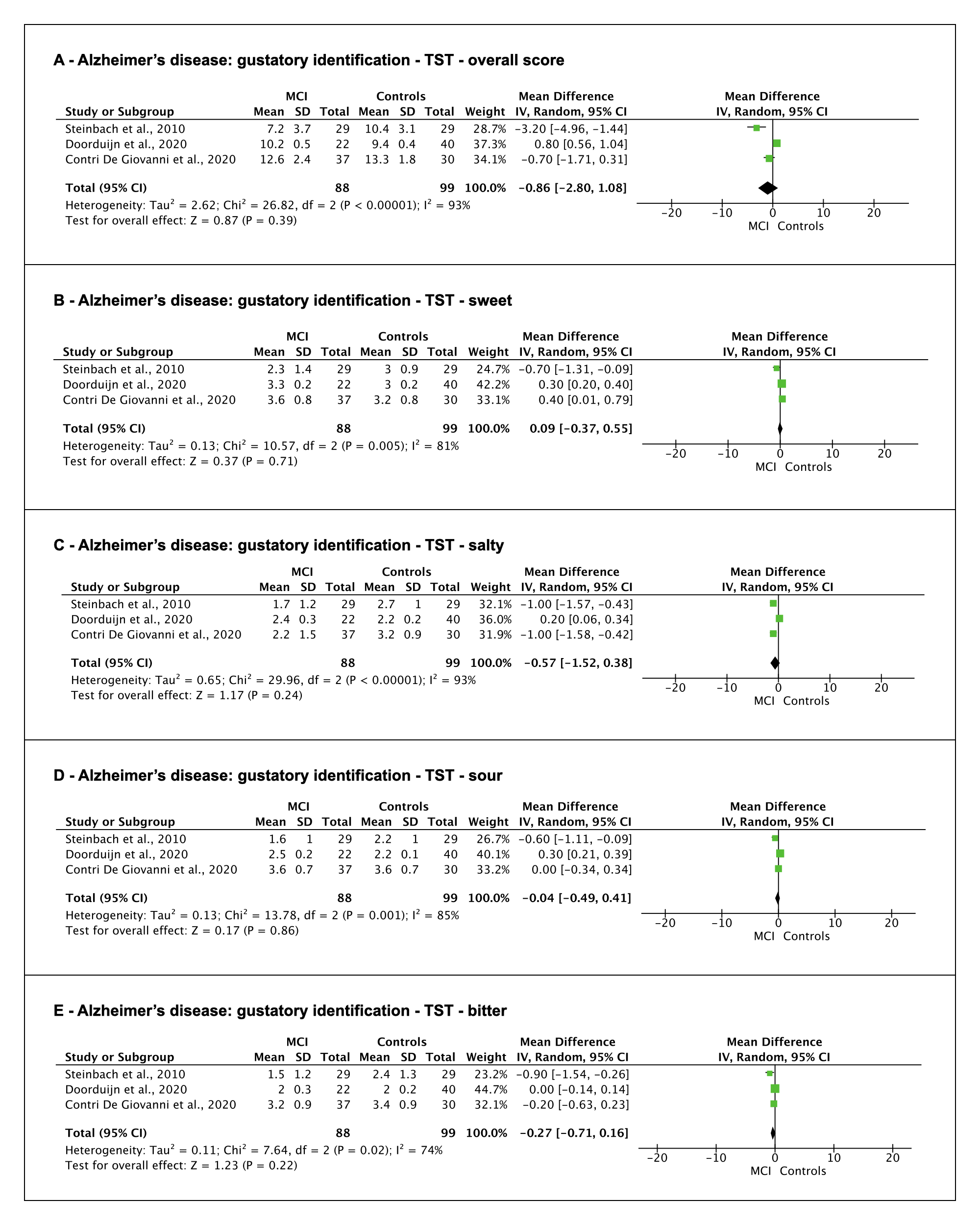

Supplement: Supplementary file 3 — Supplementary Material 3 [file 11065_2023_9578_MOESM3_ESM.jpeg]

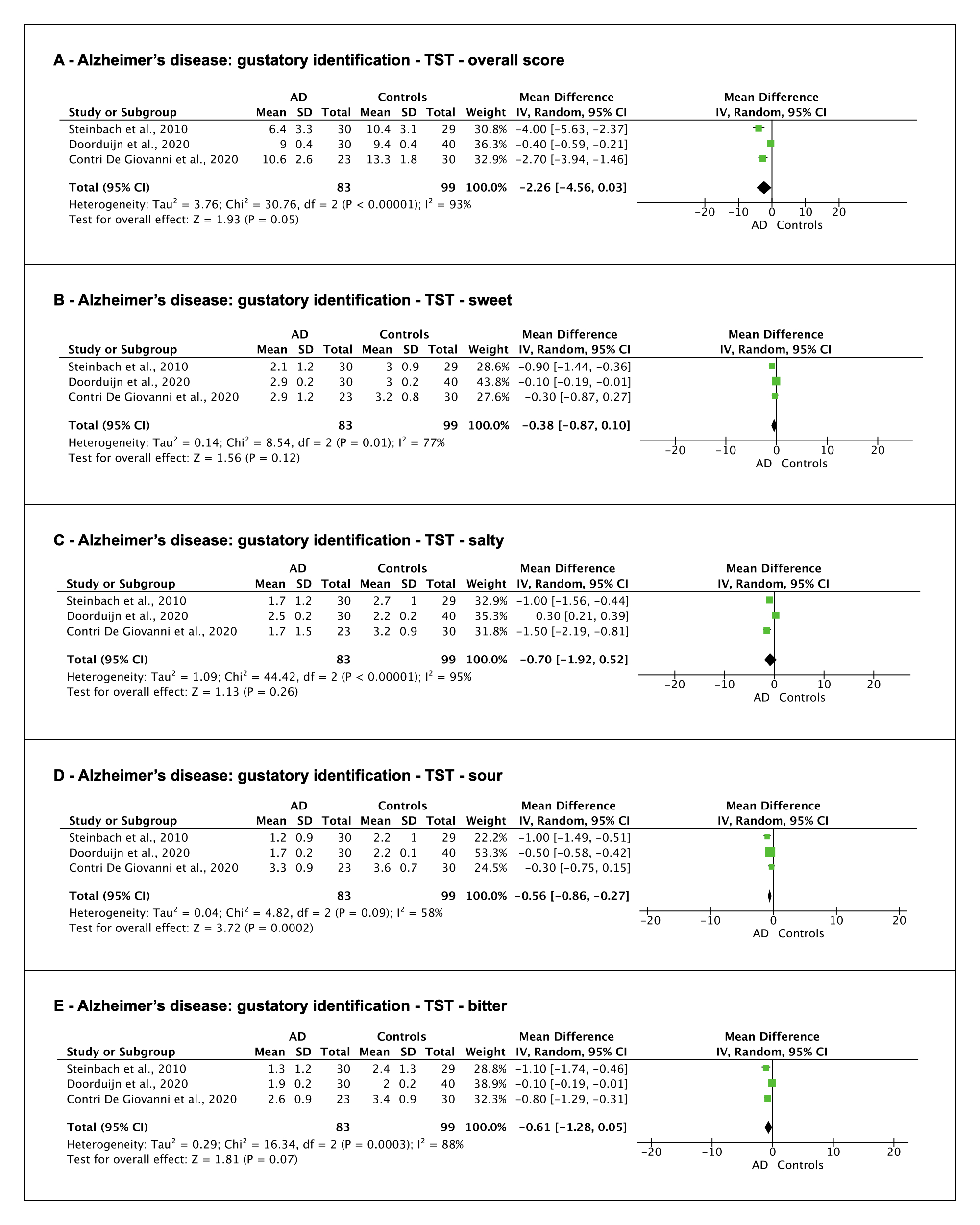

Supplement: Supplementary file 4 — Supplementary Material 4 [file 11065_2023_9578_MOESM4_ESM.jpeg]

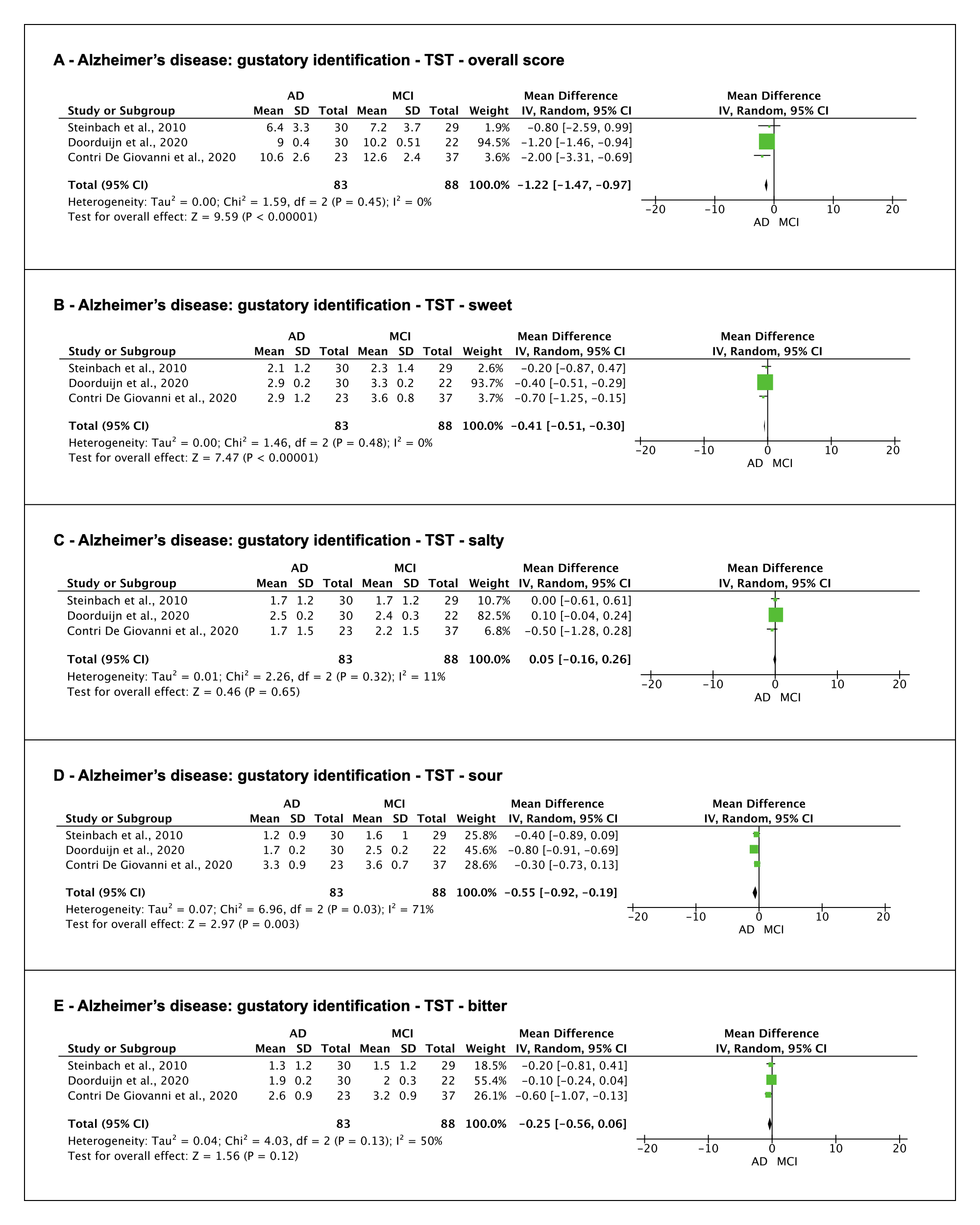

Supplement: Supplementary file 5 — Supplementary Material 5 [file 11065_2023_9578_MOESM5_ESM.jpeg]

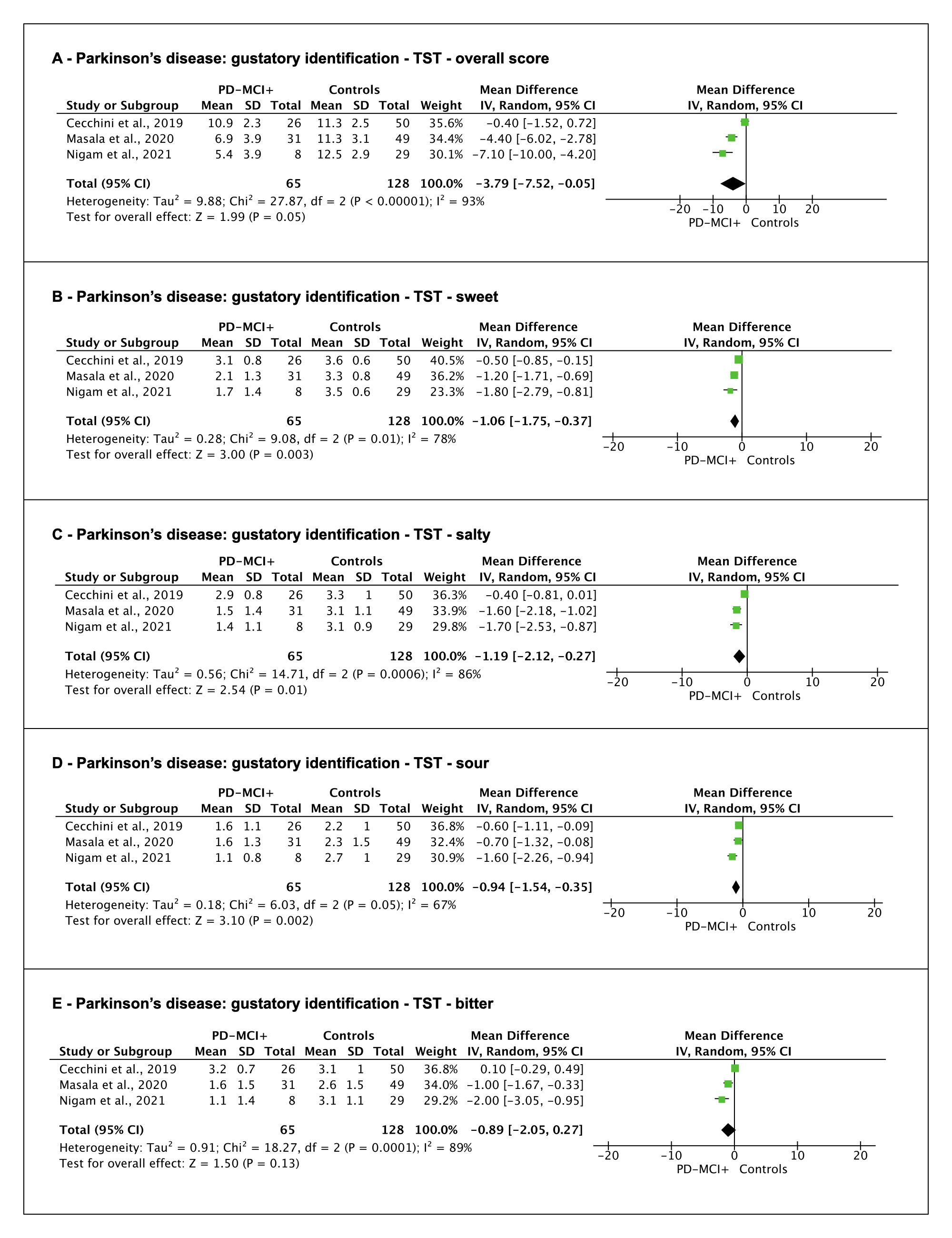

Supplement: Supplementary file 6 — Supplementary Material 6 [file 11065_2023_9578_MOESM6_ESM.jpeg]

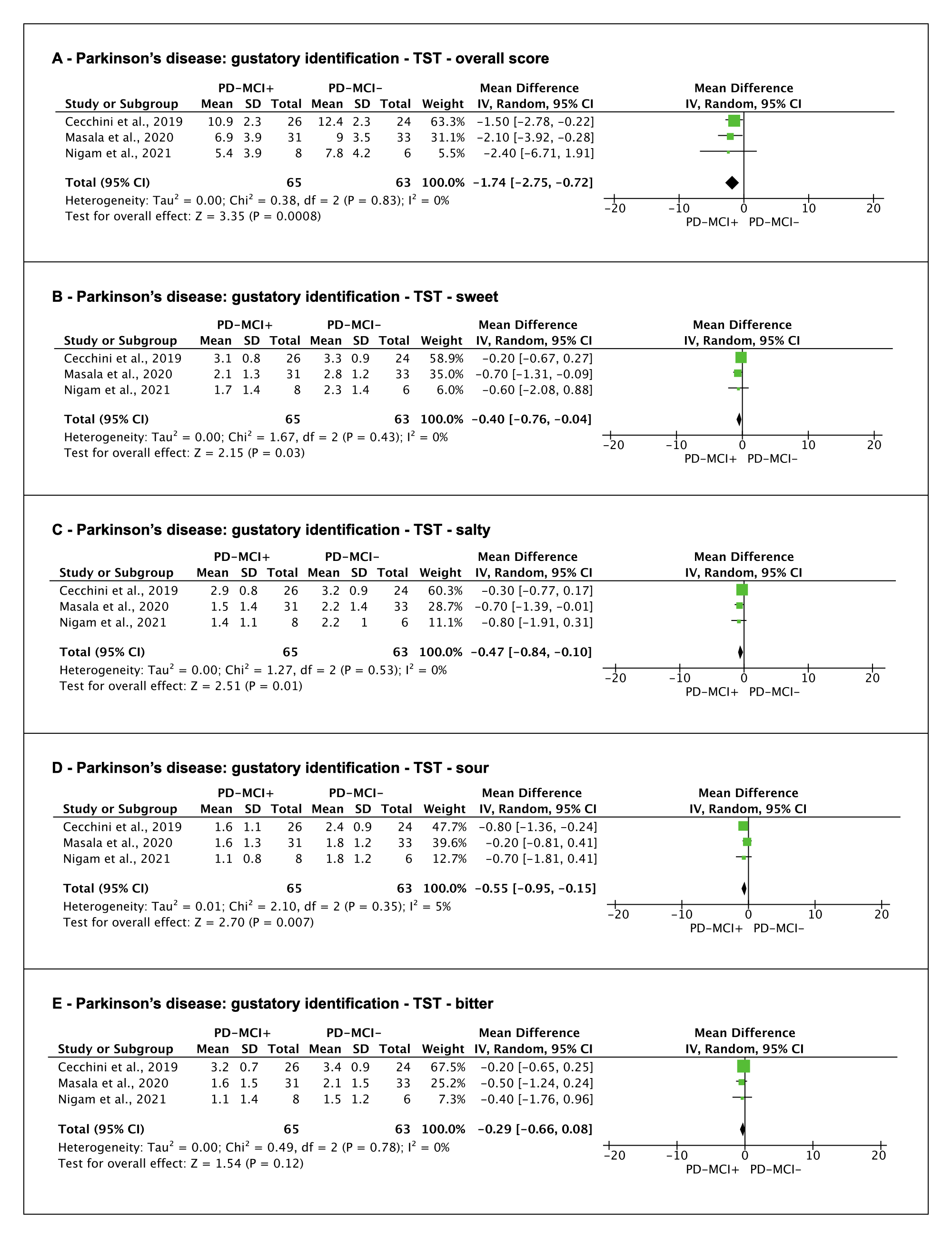

Supplement: Supplementary file 7 — Supplementary Material 7 [file 11065_2023_9578_MOESM7_ESM.jpeg]
