# Supplementary material for: The Association Between Neurocognitive Disorders and Gustatory Dysfunction: A Systematic Review and Meta-Analysis
Source: Neuropsychol Rev. 2023 Feb 20;34(1):192–213. doi: 10.1007/s11065-023-09578-3 (PMC10920407; doi:10.1007/s11065-023-09578-3)
Supplement: Supplementary file 8 — Supplementary Material 8 [file 11065_2023_9578_MOESM8_ESM.docx]

**Table S1.** Definitions of neurocognitive disorders according to the Diagnostic and Statistical Manual of Mental Disorders – 5 edition (DSM 5)

|  | **Diagnostic criteria** | **Possible causes/etiological subtypes** |
| --- | --- | --- |
| **Delirium** | 1. Disturbance in attention and awareness. 2. It develops over a short period of time, represents a change from baseline attention and awareness and fluctuates in severity during the day. 3. Possible concomitant disturbance in other cognitive domains^a^. 4. Not better explained by preexisting, established, or evolving neurocognitive disorder; not occurring exclusively in the context of severe reduced level of arousal. 5. Evidence of a direct physiological consequence of another medical condition, substance intoxication/withdrawal, exposure to a toxin, or multiple etiologies. | - Alcohol - Cannabis - Phencyclidine - Other hallucinogen - Inhalant - Opioid - Sedative, hypnotic or anxiolytic - Amphetamine/other stimulant - Cocaine - Other/unknown substance - Other medical conditions - Multiple etiologies |
| **Mild Neurocognitive Disorder** (i.e., MCI) | 1. Modest cognitive decline from a previous level of functioning in one or more domains^a^ as reported by the patient, an informant, a clinician, and/or documented by objective clinical assessment. 2. Preserved daily life activities but greater efforts, compensatory strategies or accommodation required. 3. Not occurring exclusively in the context of delirium. 4. Not better explained by another mental disorder. | - Alzheimer’s disease - Frontotemporal - Lewy Body disease - Vascular disease - Traumatic brain injury - Substance/medication use - HIV infection - Prion disease - Parkinson’s disease - Huntington’s disease - Other medical conditions - Multiple etiologies - Unspecified |
| **Major Neurocognitive Disorder** (i.e., dementia) | 1. Cognitive decline from a previous level of functioning in one or more domains^a^ as reported by the patient, an informant, a clinician, and/or documented by objective clinical assessment. 2. Interference with daily life activities. 3. Not occurring exclusively in the context of a delirium. 4. Not better explained by another mental disorder. |  |

**List of abbreviations.** MCI = Mild Cognitive Impairment. ^a^Attention, executive function, learning and memory, perceptual-motor, social cognition.

**Table S2.** Main validated procedures to assess gustatory function

| **Gustatory testing** | **Definition** | **Taste feature assessed** |
| --- | --- | --- |
| *Chemical stimuli* | | |
| Three – drop test | A series of three drops of a liquid solution, one drop containing a taste stimulus and the other two being pure tasteless water, is placed in a pseudorandomized order on the midline of the patient’s tongue. After swishing the drop into the oral cavity, the subject is asked to identify the taste quality (i.e., sweet, salty, sour, bitter). Threshold is defined at a given concentration when the subject correctly identifies the taste quality three times in a row (range: 1-9 points). | T and ID |
| Tasting tablets and wafers | Edible tablets or wafers of water and flour with six concentrations of the four basic tastes (i.e., sweet, sour, salty, bitter) are presented in a pseudorandomized order, starting with the lowest concentration. | T and ID |
| Whole – Mouth Test (WMT) | Supra-threshold taste solutions (i.e., sweet, sour, salty, bitter) sprayed in the oral cavity. | ID |
| Taste Strips Test (TST) | Filter paper strips impregnated with four concentrations of the four basic tastes (i.e., sweet, sour, salty, bitter) are placed on the tongue in a randomized order. Each correct answer yields one point (range: 0-16). | ID |
| Filter Paper Disc (FPD) | Round filter paper discs, 5 mm each, impregnated with five different concentrations of the taste qualities (i.e., sweet, sour, salty, bitter, umami) are presented in a randomized order, with bitter taste presented at last (range: 1-5 points). | T and ID |
| *Electrical stimuli* | | |
| Electrogustometry | A low intensity current of 3-400 µA is applied to the upper tongue surface by means of a 5-mm diameter stainless steel active electrode and referenced to a neck band inactive electrode. The stimulus is increased through a single staircase approach until the subject has a taste sensation. This gives a taste recognition threshold measurement, expressed in decibel (dB). The stimulus could be perceived as metallic, sour, salty or bitter. | T |
| *Gaseous stimuli* | | |
| Gustatory event-related potentials | Gustatory evoked potentials are elicited by means of gaseous stimuli (i.e., highly concentrated solutions of salty, sour, sweet, bitter and umami) presented through a gustometer. | T |

**List of abbreviations.** ID = identification; T = threshold.

**Table S3.** Results of the sensitivity analyses

|  | **Outcome and study removed for sensitivity analysis** | **K** | **N** | **Random-effect model results** | | | | **Heterogeneity** | | | | |
| --- | --- | --- | --- | --- | --- | --- | --- | --- | --- | --- | --- | --- |
|  |  |  |  | **MD** | **[95% CI]** | **Z** | ***p*** | ***Q*** | **df** | ***p*** | **τ^2^** | **I^2^ (%)** |
| **ID** | **Alzheimer’s disease (AD)** | | | | | | | | | | | |
|  | *MCI vs controls, TST (negative MD values indicate worse performance for patients than controls)* | | | | | | | | | | | |
|  | TST score  Doorduijn et al., 2020 | 2 | 125 | -1.84 | [-4.28, 0.60] | 1.48 | 0.14 | 5.86 | 2 | 0.02 | 2.59 | 83 |
|  | Sweet  Steinbach et al., 2010 | 2 | 129 | 0.31 | [0.21, 0.44] | 5.99 | **< 0.00001** | 0.24 | 2 | 0.62 | 0.00 | 0 |
|  | Salty  Doorduijn et al., 2020 | 2 | 125 | -1.00 | [-1.41, -0.59] | 4.82 | **< 0.00001** | 0.00 | 2 | 1.00 | 0.00 | 0 |
|  | Sour  Steinbach et al., 2010 | 2 | 129 | 0.20 | [-0.08, 0.48] | 1.37 | 0.17 | 2.84 | 2 | 0.09 | 0.03 | 65 |
|  | Bitter  Steinbach et al., 2010 | 2 | 129 | -0.02 | [-0.15, 0.11] | 0.28 | 0.78 | 0.74 | 2 | 0.39 | 0.00 | 0 |
|  | *AD vs controls, TST (negative MD values indicate worse performance for patients than controls)* | | | | | | | | | | | |
|  | TST score  Doorduijn et al., 2020 | 2 | 112 | -3.24 | [-4.49, -1.98] | 5.06 | **< 0.00001** | 1.54 | 2 | 0.21 | 0.30 | 35 |
|  | Sweet  Steinbach et al., 2010 | 2 | 123 | -0.11 | [-0.20, -0.01] | 2.21 | **0.03** | 0.46 | 2 | 0.50 | 0.00 | 0 |
|  | Salty  Doorduijn et al., 2020 | 2 | 112 | -1.21 | [-1.69, -0.72] | 4.90 | **< 0.00001** | 1.21 | 2 | 0.27 | 0.02 | 17 |
|  | Sour  Steinbach et al., 2010 | 2 | 123 | -0.49 | [-0.57, -0.42] | 12.61 | **< 0.00001** | 0.75 | 2 | 0.39 | 0.00 | 0 |
|  | Bitter  Doorduijn et al., 2020 | 2 | 112 | -0.91 | [-1.30, -0.52] | 4.60 | **< 0.00001** | 0.53 | 2 | 0.46 | 0.00 | 0 |
|  | *AD vs MCI, TST (negative MD values indicate worse performance for AD than MCI)* | | | | | | | | | | | |
|  | Salty  Contri De Giovanni et al., 2020 | 2 | 111 | 0.09 | [-0.05, 0.24] | 1.32 | 0.19 | 0.10 | 2 | 0.76 | 0.00 | 0 |
|  | Doorduijn et al., 2020 | 2 | 119 | -0.19 | [-0.67, 0.29] | 0.77 | 0.44 | 0.98 | 2 | 0.32 | 0.00 | 0 |
|  | Sour  Doorduijn et al., 2020 | 2 | 119 | -0.34 | [-0.67, -0.02] | 2.09 | **0.04** | 0.09 | 2 | 0.76 | 0.00 | 0 |
|  | Bitter  Contri De Giovanni et al., 2020 | 2 | 111 | -0.11 | [-0.25, 0.04] | 1.47 | 0.14 | 0.10 | 2 | 0.76 | 0.00 | 0 |
|  | Doorduijn et al., 2020 | 2 | 119 | -0.45 | [-0.83, -0.07] | 2.33 | **0.02** | 1.03 | 2 | 0.31 | 0.00 | 3 |
|  | **Parkinson’s disease** | | | | | | | | | | | |
|  | *PD-MCI+ vs controls, TST Test (negative MD values indicate worse performance for patients than controls)* | | | | | | | | | | | |
|  | TST score  Cecchini et al., 2019 | 2 | 117 | -5.47 | [-8.06, -2.88] | 4.14 | **< 0.0001** | 2.53 | 2 | 0.11 | 2.21 | 61 |
|  | Sweet  Cecchini et al., 2019 | 2 | 117 | -1.34 | [-1.84, -0.84] | 5.26 | **< 0.00001** | 1.11 | 2 | 0.29 | 0.02 | 10 |
|  | Salty  Cecchini et al., 2019 | 2 | 117 | -1.63 | [-2.11, -1.16] | 6.72 | **< 0.00001** | 0.04 | 2 | 0.85 | 0.00 | 0 |
|  | Sour  Nigam et al., 2021 | 2 | 156 | -0.64 | [-1.03, -0.25] | 3.20 | **0.001** | 0.06 | 2 | 0.81 | 0.00 | 0 |
|  | Bitter  Cecchini et al., 2019 | 2 | 117 | -1.42 | [-2.38, -0.45] | 2.87 | **0.04** | 2.47 | 2 | 0.12 | 0.30 | 59 |

**List of abbreviations.** CI = confidence interval; ID = identification; K = number of studies; MCI = mild cognitive impairment; MD = mean difference; N = number of participants; PD-MCI+ = Parkinson’s disease with mild cognitive impairment; TST = Taste Strips Test. *P* values ≤ 0.05 are reported in bold type.

**Table S4.** Studies assessing both olfactory and taste function

| **Ref.** | **Etiology** | **NCD severity** | **Olfactory function** | **Taste function** |
| --- | --- | --- | --- | --- |
| Murphy et al., 1990 | AD | Major NCD | I | U |
| Schiffman et al., 1990 | AD, VaD | Major NCD | I | I |
| Murphy et al., 1999 | AD | Major NCD | I | U |
| Schiffman et al., 2002 | AD | Mild, major NCD | I | I |
| Lang et al., 2006 | AD, FTD, PD | Major NCD | I | I |
| Steinbach et al., 2010 | AD | Mild, major NCD | I | I |
| Brion et al., 2015 | KS | Major NCD | I | I |
| Kouzuki et al., 2018 | AD | Mild, major NCD | I | U |
| Cecchini et al., 2019 | PD | Mild NCD | I | I |
| Churnin et al., 2019 | AD | Mild, major NCD | I | I |
| Doorduijn et al., 2020 | AD | Mild, major NCD | I | U |
| Masala et al., 2020 | PD | Mild NCD | I | I |
| Nigam et al., 2021 | PD | Mild NCD | I | I |

**List of abbreviations.** AD = Alzheimer’s disease; FTD = Fronto-temporal disease; I = impaired; KS = Korsakoff syndrome; NCD = neurocognitive disorder; PD = Parkinson’s disease; U = unimpaired; VaD = vascular dementia.
